# Evidence of two-dimensional flat band at the surface of antiferromagnetic kagome metal FeSn

**DOI:** 10.1038/s41467-021-25705-1

**Published:** 2021-09-15

**Authors:** Minyong Han, Hisashi Inoue, Shiang Fang, Caolan John, Linda Ye, Mun K. Chan, David Graf, Takehito Suzuki, Madhav Prasad Ghimire, Won Joon Cho, Efthimios Kaxiras, Joseph G. Checkelsky

**Affiliations:** 1 Department of Physics, Massachusetts Institute of Technology, Cambridge, MA USA; 2 Frontier Research Institute for Interdisciplinary Sciences and Institute for Materials Research, Tohoku University, Miyagi, Japan; 3 Department of Physics and Astronomy, Center for Materials Theory, Rutgers University, Piscataway, NJ USA; 4 National High Magnetic Field Laboratory, LANL, Los Alamos, NM USA; 5 National High Magnetic Field Laboratory, Tallahassee, FL USA; 6 Central Department of Physics, Tribhuvan University, Kirtipur, Kathmandu Nepal; 7 Leibniz Institute for Solid State and Materials Research, IFW Dresden, Dresden, Germany; 8 Samsung Advanced Institute of Technology (SAIT), Suwon-si, Gyeonggi-do Korea; 9 Department of Physics, Harvard University, Cambridge, MA USA; 10 Present Address: National Institute of Advanced Industrial Science and Technology, Tsukuba, Japan; 11 Present Address: Department of Applied Physics, Stanford University, Stanford, CA USA; 12 Present Address: Department of Physics, Toho University, Chiba, Japan

**Keywords:** Electronic properties and materials, Surfaces, interfaces and thin films, Topological insulators

## Abstract

The kagome lattice has long been regarded as a theoretical framework that connects lattice geometry to unusual singularities in electronic structure. Transition metal kagome compounds have been recently identified as a promising material platform to investigate the long-sought electronic flat band. Here we report the signature of a two-dimensional flat band at the surface of antiferromagnetic kagome metal FeSn by means of planar tunneling spectroscopy. Employing a Schottky heterointerface of FeSn and an n-type semiconductor Nb-doped SrTiO_3_, we observe an anomalous enhancement in tunneling conductance within a finite energy range of FeSn. Our first-principles calculations show this is consistent with a spin-polarized flat band localized at the ferromagnetic kagome layer at the Schottky interface. The spectroscopic capability to characterize the electronic structure of a kagome compound at a thin film heterointerface will provide a unique opportunity to probe flat band induced phenomena in an energy-resolved fashion with simultaneous electrical tuning of its properties. Furthermore, the exotic surface state discussed herein is expected to manifest as peculiar spin-orbit torque signals in heterostructure-based spintronic devices.

## Abstract (web summary)

Transition metal kagome compounds have been shown to host flat bands in their bulk electronic spectrum. Here, using planar tunnelling spectroscopy supported by first-principles calculations, the authors report the signature of a novel type of flat band at the surface of antiferromagnetic kagome metal FeSn.

## Introduction

When the electron–electron interaction becomes the dominant energy scale in a condensed matter system, a variety of interaction-driven quantum phases are expected to arise. One way to design a system with interaction energy larger than the kinetic energy of individual constituents is to confine electrons into a flat, dispersionless band in momentum space. Historically, flat bands have been realized when inherently localized atomic orbitals constitute a periodic lattice (i.e., *f*-electron bands)^1,2^ or when magnetic field traps electrons to quantized cyclotron orbits (i.e., Landau levels in quantum Hall phases)^3,4^. More recently, there has been a growing interest in constructing a generalized lattice model that can universally produce flat bands through destructive phase interference of electronic hopping, even in the absence of compact atomic orbitals or high magnetic field^5–12^.

**Fig. 1:**
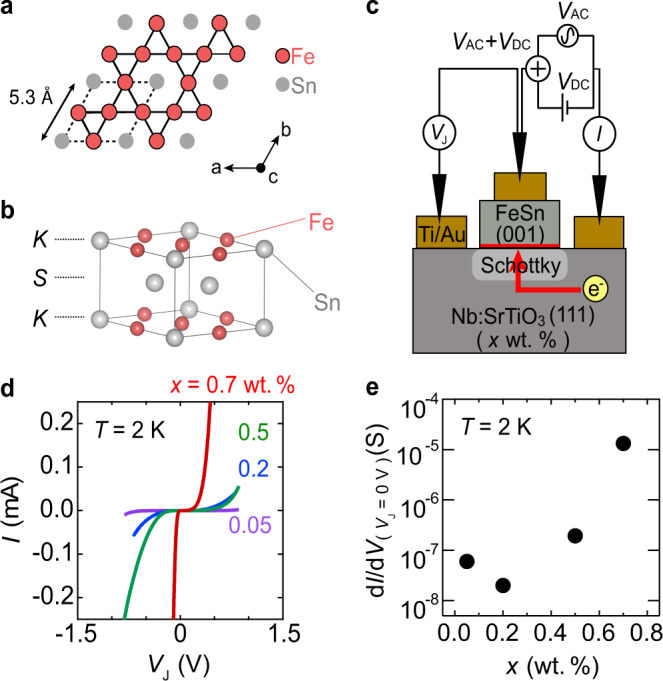
Tunneling measurements across FeSn/Nb:STO heterointerfaces. **a** Top view of the Fe kagome layer (Fe_3_Sn). Dashed lines delineate the crystallographic unit cell. **b** Crystal structure of FeSn, consisting of kagome layers (*K*) and Sn honeycomb (stanene) layers (*S*) stacked along the *c*-direction. **c** Schematic of the three-terminal planar Schottky tunneling measurement setup. AC (*V*_AC_) and DC (*V*_DC_) voltages are applied onto the tunneling electrode and the junction voltage (*V*_J_) and the tunnel current (*I*) are measured. **d** Current-voltage (*I*–*V*) characteristics of FeSn/Nb:STO junctions with different Nb concentrations, all measured at temperature *T* = 2 K. **e** Nb concentration-dependent zero bias differential tunneling conductance (d*I*/d*V*(*V*_J_ = 0 V)) at *T* = 2 K.

The kagome lattice, a two-dimensional hexagonal network of corner-sharing triangles (Fig. 1a), is one example of a lattice model that accommodates a flat band. In addition, the *D*_6_ point group symmetry in the kagome lattice, similar to graphene, engenders a linearly dispersing Dirac band, which with the inclusion of spin-orbit coupling and non-zero magnetization hosts a topologically nontrivial Chern gap^13–20^. Following decades of theoretical predictions, recent angle-resolved photoemission spectroscopy measurements on transition metal kagome compounds have shown that certain features in the two-dimensional electronic structure of a single kagome layer manifest largely unperturbed in their three-dimensional electronic structures^13,21,22^. However, it was also observed that the physics of a single kagome layer can be significantly altered in other kagome compounds with sufficiently large inter-layer hybridization^23–26^, suggestive of the importance of strong electronic two-dimensionality in connecting to the original lattice model.

Though real crystals inevitably harbor non-zero inter-layer couplings (i.e., orbital hybridization, charge transfer, magnetic interaction), it has been pointed out from a number of three-dimensional systems that their surfaces offer a unique venue that connects to the character of their parent two-dimensional unit cells. For instance, from the surface of bismuth single crystals, a two-dimensional electron gas and quantum Hall wavefunction imprinting the crystallographic symmetry of a single bismuthene layer have been observed^27–31^. In addition, the Chern insulating phase has been predicted at the kagome-terminated surface of Cs_2_LiMn_3_F_12_, a ferromagnetic insulator containing completely filled kagome-derived bands. In the absence of charge-donating adlayers, the local chemical potential of the bare kagome network at the surface was expected to cross the Dirac mass gap. The Chern insulating phase has also been realized but through a different mechanism at the surface of an antiferromagnetic topological insulator^32–34^. There, the local magnetic field distinct from the global mean field stabilized a distinguished phase at the surface.

In this study, we investigate a flat band at the surface of antiferromagnetic kagome metal FeSn using planar tunneling spectroscopy. FeSn consists of an alternating stack of two-dimensional Fe kagome layers and two-dimensional Sn honeycomb (stanene) layers (Fig. 1a, b). It is known to develop a type-II antiferromagnetic order below *T*_N_ = 365 K, in which the Fe spin moments align ferromagnetically within a single kagome plane but antiferromagnetically from those in the neighboring kagome planes^35,36^. Along with the magnetic degrees of freedom inter-weaved therein, its characteristic layered crystal structure makes FeSn an ideal platform to explore the interplay of the kagome lattice with the honeycomb lattice at the surface with different types of atomic terminations. For surface-sensitive spectroscopy, we used molecular beam epitaxy (MBE) to synthesize epitaxial films of FeSn on lattice-matched n-type degenerate semiconductor Nb-doped SrTiO_3_ (Nb:STO). Combining tunneling spectroscopy and first-principles calculations, we find that the observed signals constitute signatures consistent with a two-dimensional flat band originating from the spin-polarized surface kagome-stanene bilayer unit cell.

## Results

### Planar tunneling spectroscopy measurements

Epitaxial thin films of FeSn were deposited on Nb:STO with varying Nb concentrations (*x* = 0.05, 0.2, 0.5, 0.7 wt.%) by MBE^37^ (see Methods). X-ray diffraction measurements confirmed the formation of (001) oriented FeSn films with in-plane crystallographic orientation epitaxially locked to that of Nb:STO (see Supplementary Note 3, 20). Cross-sectional transmission electron microscopy (TEM) and electron energy loss spectroscopy measurements corroborate that the films are highly crystalline down to the interface, which itself is comprised of the Fe kagome layer and Ti-rich termination layer of Nb:STO (see Supplementary Note 21). The Neel temperature of the films was found to be consistent with that of bulk single-crystal FeSn, which exhibits a type-II antiferromagnetic spin structure^35–37^. When the two materials come in contact, a depletion layer is formed at the Schottky interface, creating an insulating barrier useful for tunneling measurements^38–40^. Figure 1c shows a schematic of the measurement setup in the three-terminal configuration, consisting of the tunnel (middle), current (right), and reference (left) electrodes. Upon applying a voltage on the tunnel electrode, a tunnel current flows between the tunnel and current electrodes across the Schottky barrier. Simultaneously, the reference potential with respect to the reference electrode was measured in order to precisely estimate the junction voltage *V*_J_. As the tunnel current is determined by the total number of electronic states which electrons can tunnel into, the differential tunneling conductance d*I*/d*V* encodes the energy-resolved density of states (DOS) of FeSn overlaid onto a monotonic background signal arising from, e.g., energy-dependent DOS of the tunnel electrode^40–46^. We note that in the regime where the tunnel electrode has a small Fermi energy (i.e., *E*_F,electrode_ < < ∣*V*_J_∣) when *V*_J_ < 0 the observed d*I*/d*V* is a direct measure of the intrinsic DOS spectrum (the case we will investigate primarily here); this is contrary to the case of *V*_J_ > 0 where the absence of electronic states below the electrode’s conduction band edge can make such a direct analysis more difficult (see Supplementary Note 23). Scanning tunneling spectroscopy (STS) studies on bulk single crystals have proven their extreme sensitivity to the local electronic states at the cleaved surface, enabling atomic termination dependent DOS characterizations^47,48^. In the case of Schottky tunneling, the tunneling conductance is expected to be most sensitive to the bottom-most layer of FeSn at the Schottky interface with Nb:STO, FeSn, and the Schottky barrier each serving the role of the tip, sample, and vacuum in STS, respectively.

Figure 1d shows the current-voltage (*I*–*V*) characteristics of FeSn/Nb:STO junctions with different Nb concentrations, all acquired at temperature *T* = 2 K. All of the *I*–*V* traces show non-linear behavior, reflecting the tunneling transport process across these junctions. Typical tunneling resistance of the FeSn/Nb:STO (*x* = 0.5 wt.%) junction at *T* = 2 K and *V*_J_ = 0 V was >1 MΩ with minor variance between different devices. This is much larger than the 7 Ω resistance observed across the Ti/Nb:STO ohmic junctions on the same sample (see Supplementary Note 2). These suggest the presence of a depletion layer at the FeSn/Nb:STO interface. Figure 1e shows the exponential growth of the zero bias differential tunneling conductance at *T* = 2 K for Nb concentration from *x* = 0.05 wt.% to *x* = 0.7 wt.%. We ascribe this to the cooperative action of increased carrier density (*N*_d_) and suppressed dielectric permittivity (*ϵ*) in highly doped Nb:STO dramatically shortening the depletion layer width *W*_d_ = $\sqrt{\frac{2\epsilon {{\Delta }}{{{\Psi }}}_{{{{{{{{{{\rm{WF}}}}}}}}}}}}{q{N}_{d}}}$ at the Schottky interface, where *q* is elementary charge and ΔΨ_WF_ is work function difference between FeSn and Nb:STO^49–51^. Fermi level pinning and its subsequent screening from Nb-doping may also be present but represent a negligible contribution compared with that observed (see Supplementary Note 22). Consistent with our observation, recent studies have shown that *W*_d_ ~ 5 nm in Pt/Nb:STO (*x* = 0.7 wt.%) Schottky junctions^52^, in contrast with *W*_d_ > 100 nm in metal/Nb:STO Schottky junctions with lower Nb concentrations^38,44,53,54^.

### Temperature-dependent tunneling in FeSn/Nb:STO (*x* = 0.5 wt.%)

**Fig. 2:**
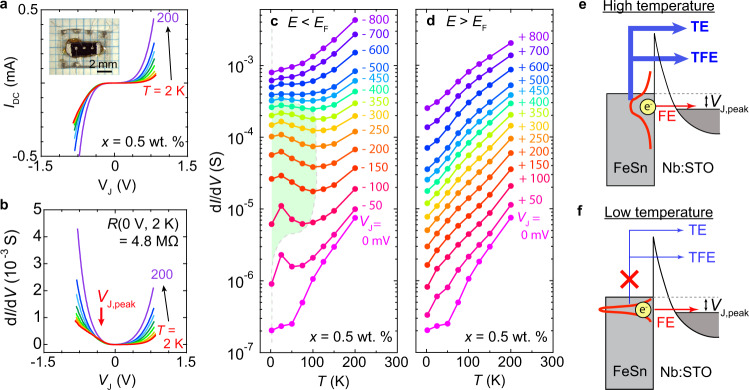
Temperature-dependent tunneling in a *x* = 0.5 wt.% junction. **a***I*–*V* characteristics and **b** d*I*/d*V* spectra at different temperatures for the FeSn/Nb:STO junction with *x* = 0.5 wt. %. The measurements were taken at *T* = 2, 25, 50, 75, 100, 125, 150, 200 K. The inset in **a** is an optical micrograph of the measuring device. The red arrow in **b** marks *V*_J,peak_, the position of the broad peak in d*I*/d*V* at low temperature. **c**, **d** Temperature-dependent d*I*/d*V* for negative and positive *V*_J_ for the *x* = 0.5 wt.% junction. The green-shaded area denotes the region in which d*I*/d*V* increases as temperature decreases. **e**, **f** Schematic of the tunneling mechanisms across the Schottky barrier. Non-resonant thermionic emission (TE) and thermionic field emission (TFE) processes dominate in the high-temperature regime, whereas resonant field emission (FE) process through the barrier dominates in the low-temperature regime.

Figure 2a, b show *I*–*V* curves and d*I*/d*V* spectra, respectively, at different temperatures for the FeSn/Nb:STO (*x* = 0.5 wt.%) junction (the device micrograph is shown in Fig. 2a inset). The overall tunneling conductance, as revealed from both *I* and d*I*/d*V*, gradually increases as *T* increases, owing to the exponential growth of thermionic emission (TE) and thermionic field emission (TFE) contributions (Fig. 2e)^50,53^. This resembles the behavior of a conventional Schottky junction in which enhanced thermal activation probability of electrons at high-temperature boosts the junction current^55^. We note that TE and TFE are non-resonant processes (*E*_initial_ ≠ *E*_final_, where *E*_initial_ and *E*_final_ are the energy of electrons before and after the tunneling, respectively) and therefore the resulting broadened d*I*/d*V* spectra at high temperatures obscure fine DOS features of FeSn.

When TE and TFE are sufficiently suppressed at low *T*, the d*I*/d*V* spectra reveal an anomalous behavior beyond that expected for conventional Schottky barriers. Figure 2c, d show the temperature-dependent d*I*/d*V* for negative and positive *V*_J_, respectively. Although d*I*/d*V* over the entire range of *V*_J_ decreases exponentially as *T* decreases, within the finite range −400 mV < *V*_J_ < −100 mV, the exponential suppression of d*I*/d*V* at *T* > 100 K gives way to an upturn in d*I*/d*V* at *T* < 100 K. This feature is also manifested as a broad peak in the d*I*/d*V* spectra at *T* = 2 K (*V*_J,peak_ = −250 mV) that eventually broadens and diminishes at higher *T*. Qualitatively, this feature can be understood as a combination of an anomalous enhancement of d*I*/d*V* in the negative bias range with the conventional rectifying behavior of Schottky diodes in the positive bias range. The enhancement in d*I*/d*V* at lower *T* suggests a dominant field emission (FE) contribution to the tunneling conductance for *T* < 100 K (Fig. 2f). FE is a resonant process (*E*_initial_ = *E*_final_) that becomes more pronounced in lower *T* when thermal band broadening in FeSn and inelastic scattering events within the tunnel barrier both diminish. The upturn in d*I*/d*V* around *V*_J,peak_ = −250 mV for *T* < 100 K suggests high DOS concentrated at this energy in FeSn, manifested more clearly as the FE dominates the tunneling process.

### Nb concentration-dependent tunneling and surface electronic structure

**Fig. 3:**
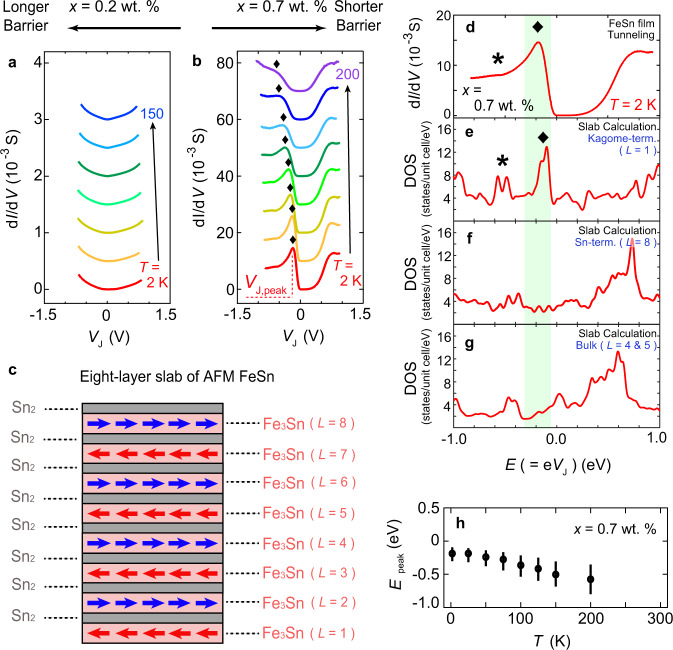
Tunneling in *x* = 0.2, 0.7 wt.% junctions. d*I*/d*V* spectra at different temperatures for FeSn/Nb:STO junctions with **a** *x* = 0.2 wt.% and **b** *x* = 0.7 wt.%. The measurements were taken at *T* = 2, 25, 50, 75, 100, 125, 150, 200 K. Each curve is offset vertically by an equal amount with respect to the *T* = 2 K trace for clarity. For the *x* = 0.7 wt.% junction in **b**, the positions of the peak in d*I*/d*V* are marked with diamonds. **c** Schematic of the eight-layer slab of antiferromagnetic FeSn. **d** d*I*/d*V* spectrum for the *x* = 0.7 wt.% junction at *T* = 2 K. Energy-dependent DOS spectra at **e** the kagome-terminated surface, **f** the Sn-terminated surface, and **g** the bulk of the eight-layer FeSn slab with the antiferromagnetic spin configuration. The green-shaded box across **d**–**g** denotes the energy window in which the peak feature in d*I*/d*V* was observed. Diamonds and asterisks mark the positions of noticeable features that correlate between **d** and **e**. **h** Temperature-dependent d*I*/d*V* peak positions (*E*_peak_) (circles) and corresponding full widths at half maximum (FWHM) (vertical bar), extracted from **b**.

To elucidate the origin of the anomaly in d*I*/d*V* seen in Fig. 2, we investigate the tunneling characteristics of two junctions with different Nb concentrations: d*I*/d*V* spectra for *x* = 0.2 wt.% and *x* = 0.7 wt.% are shown in Fig. 3a and b, respectively. The prominent peak in d*I*/d*V* is resolved ~*V*_J,peak_ = −180 mV at *T* = 2 K for *x* = 0.7 wt.%, whilst for *x* = 0.2 wt.%, the feature is absent. We hypothesize that the 20-fold enhancement of the overall tunneling conductance from *x* = 0.2 wt.% to *x* = 0.7 wt.% originates from the difference in the depletion layer widths. The peak in d*I*/d*V* at *T* = 2 K for the *x* = 0.7 wt.% junction occurs at a similar energy range as the broad peak in d*I*/d*V* for the *x* = 0.5 wt.% junction (*V*_J,peak_ = −250 mV, Fig. 2c), indicating a common origin of the two conductance anomalies. If originating from a peak in the DOS of FeSn, it would be expected that the associated peak feature in d*I*/d*V* would become less prominent for junctions with lower Nb concentrations, as electron tunneling across the thicker depletion layer involves more inelastic scattering events. This is in fact what is observed as the Nb concentration is changed from *x* = 0.7 wt.% to *x* = 0.2 wt.%. Therefore, we conclude that the enhancement in d*I*/d*V* at *V*_J,peak_ = −180 mV originates from a large, narrowly peaked DOS at this energy in FeSn. We point out additionally that Ti-3*d* *t*_2g_-derived conduction bands of Nb:STO harbor a relatively featureless DOS spectrum in the energy range of interest here and therefore are not expected to generate any prominent spectral feature. Although oxygen vacancies with a few % concentration (below the detectable limit) may be present in Nb:STO, oxygen-vacancy-induced states typically occur below the conduction band edge of Nb:STO^56–59^. The effects of these states are to broaden the d*I*/d*V* features by inelastic tunneling and potentially give rise to additional d*I*/d*V* peaks in the *V*_J_ > 0 range. However, given the current experimental configuration, these defect states inside the bandgap of Nb:STO are not expected to generate any feature to d*I*/d*V* in the *V*_J_ < 0 range (see Supplementary Note 1, 23).

In order to directly correlate the features in the tunneling spectra to those in the DOS of FeSn, we performed first-principles electronic structure calculations of a slab containing eight crystallographic unit cells of FeSn (1 ≤ *L* ≤ 8), where *L* denotes the layer index (Fig. 3c, see Methods). Here, we recall that d*I*/d*V* reflects DOS of FeSn for *V*_*J*_ < 0 except for a monotonic background signal (see Supplementary Note 23). The slab has Fe kagome layers (Fe_3_Sn) at each layer index site with stanene layers (Sn_2_) inserted between (Fig. 3c). This structure terminates with the Fe kagome layer on one surface (*L* = 1) and the stanene layer on the other surface (*L* = 8). The d*I*/d*V* spectrum for the *x* = 0.7 wt.% junction at *T* = 2 K (Fig. 3d) is compared with the calculated DOS spectra at the kagome-terminated surface (Fig. 3e), the Sn-terminated surface (Fig. 3f), and the bulk part of the slab (Fig. 3g). Within the energy range of the d*I*/d*V* peak (green-shaded box across Fig. 3d–g), the kagome-terminated surface hosts a clear peak in DOS at *E* = −125 meV (diamonds in Fig. 3d, e) whereas the other two do not manifest any pronounced feature. In addition, a shoulder-like feature in d*I*/d*V* is observed at *V*_J_ = −560 mV nearby the satellite peak in DOS at the kagome-terminated surface at *E* = −525 meV (asterisks in Fig. 3d, e). These suggest that the major features in the tunneling spectra including the peak at *V*_J,peak_ = −180 mV originate from the electronic states at the kagome-terminated surface of FeSn.

Figure 3h shows the temperature dependence of d*I*/d*V* peak positions and full widths at half maximum of the *x* = 0.7 wt.% junction. In addition to peak broadening, the peak position gradually shifts from *V*_J,peak_ = −180 mV at *T* = 2 K to *V*_J,peak_ = −560 mV at *T* = 200 K. Though the triangular shape of the tunnel barrier and non-linear dielectric properties of SrTiO_3_ (STO) in metal/Nb:STO Schottky junction is known to displace the energy axis of the tunneling spectra from the actual DOS spectra by ~10 mV at high temperature, this is insufficient to explain the large shift of ∣Δ*V*_J,peak_∣ = 380 mV (see the model calculation for the tunneling spectra in Supplementary Note 7, 8, 24). Instead, we hypothesize that this shift originates from the modulation of the surface band structure. We examine this more thoroughly in the following sections by considering spin polarization-dependent band reconstruction.

### Surface accommodation of the spin-polarized two-dimensional flat band

**Fig. 4:**
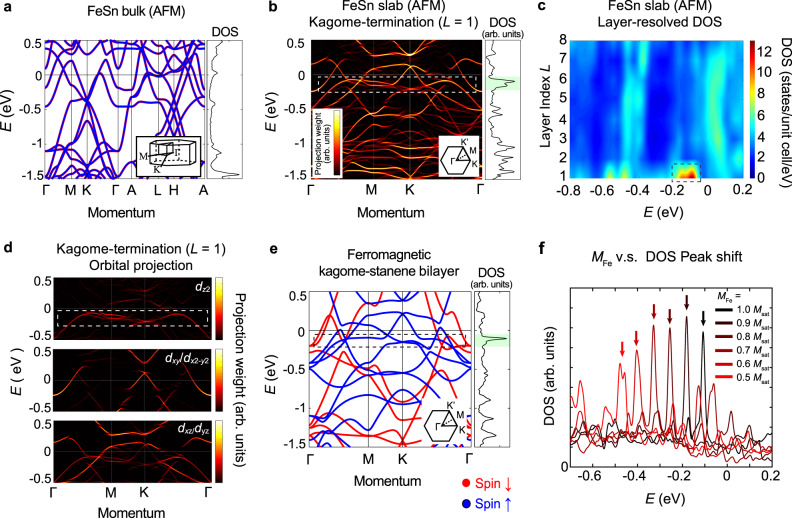
Spin-polarized surface flat band in FeSn. First-principles band structure calculations and DOS spectra of **a** bulk FeSn in the antiferromagnetic phase and **b** kagome-terminated surface of the eight-layer FeSn slab with the antiferromagnetic spin configuration. The intensity denotes Fe *d*-orbital projection weights in arbitrary units (arb. units). Insets in **a**, **b** show schematics of corresponding Brillouin zones. Spin down/up bands are color-coded with red/blue in **a**. The DOS peak and the flat band in **b** are marked with the green-shaded box and the dashed line, respectively. **c** Layer-resolved DOS color map in the eight-layer slab. **d** Orbital projected band structure of **b**. **e** Band structure and DOS spectrum of a ferromagnetic kagome-stanene bilayer. **f** Average Fe spin moment (*M*_Fe_)-dependent DOS spectra of the ferromagnetic kagome-stanene bilayer. The DOS peak associated with the flat band shifts to higher binding energy as *M*_Fe_ depolarizes.

To understand the origin of the DOS peak at the surface, it is instructive to compare the bulk and surface electronic structures. Figure 4a shows the band structure of bulk FeSn in the antiferromagnetic phase. Some of the essential features of the two-dimensional kagome network appear intact in the three-dimensional band structure of bulk FeSn, including the Dirac point at the *K*-point near *E* ~ −460 meV and the flat band centered around *E* ~ 600 meV (only the bottom band edge visible in Fig. 4a). The latter was identified from previous studies to have *d*_*xz*_/*d*_*yz*_ and *d*_*xy*_/*d*_*x*2−*y*2_ orbital character^21,22,60,61^ and is responsible for the prominent peak in DOS around *E* ~ 600 meV in Fig. 3g. Turning to the kagome-terminated surface band structure (Fig. 4b), near the energy at which the DOS peak is expected, a highly non-dispersive band is observed (enclosed with the dashed line in Fig. 4b). The layer-resolved DOS color map (Fig. 4c) also corroborates the existence of the surface-localized (*L* = 1) flat band with high DOS concentrated at that energy and the orbital projection analysis (Fig. 4d) reveals that the major contribution to the surface flat band comes from *d*_*z*2_ orbital, distinct from the orbital character of the bulk flat band. Considering the vertically elongated shape of *d*_*z*2_ orbital and its hybridization with Sn *p* orbitals in neighboring stanene layers, it is expected to gain a sizeable dispersion along *z* when placed inside the bulk, resulting in a dilution of the spectral weight in energy. However, at the surface, the translational invariance is broken and *k*_*z*_-dispersion is quenched, thereby allowing a surface state non-dispersive within the *ab*-plane as well as along the *c* axis. This demonstrates an approach to engineering a high degree of band flattening along all three directions for *d*-orbital based flat bands that may prove useful for a wide variety of systems.

To gain further insight into the surface flat band, we calculated the two-dimensional band structure of a ferromagnetic kagome-stanene bilayer, which constitutes half of the magnetic unit cell of FeSn (Fig. 4e). The band structure of the ferromagnetic kagome-stanene bilayer reasonably matches that of the kagome-terminated surface (Fig. 4b). It also exhibits a spin-polarized non-dispersive band (enclosed with the dashed line in Fig. 4e) that nearly coincides in shape and energy with the surface flat band in Fig. 4b. The resemblance between the two reflects the layered crystal structure of FeSn (Fig. 1b) in which hybridization between the consecutive kagome layers is suppressed by the stanene layers. This as a result allows FeSn band structure to be well described by the minimal constituent of the kagome-stanene bilayer. We also find that the intrinsically inversion asymmetric kagome-stanene bilayer is most precisely represented by the kagome layer at the surface (or Schottky interface) that neighbors a stanene on one side and vacuum (or Nb:STO) on the other side. However, kagome layers in the bulk, being sandwiched by two stanene layers, are situated in an inversion symmetric environment and therefore give rise to the band structure deviated from that of the kagome-stanene bilayer (see Supplementary Note 15). We note that while the stanene layers play an important structural role as well as control the local symmetry environment, the presence of Sn in both the stanene and kagome layers introduces three-dimensional hopping pathways that preclude the observation of two-dimensional stanene bands (which itself might be mitigating with isolation of the stanene layers)^62^.

Further analyses reveal that the complex interaction of the kagome layer and the stanene layer under an inversion asymmetric environment has an important influence on the flat band in the kagome-stanene bilayer. By considering continuous evolution of band structure in fictitious bilayers with variable inter-layer spacing *z*_K−S_, we find that the bands originally expected in the kagome monolayer limit (*z*_K−S_ ≫ 2.24 Å) gradually deform into the ones in the kagome-stanene bilayer (*z*_K−S_ = 2.24 Å) as *z*_K−S_ shrinks. In particular, the Fermi velocity *v*_F_ of one of the Dirac bands in the monolayer limit decreases by more than fivefold in the bilayer limit, as a result generating the *d*_*z*2_ orbital derived bilayer flat band with a Dirac-like crossing squeezed within the highly flattened dispersion (see Supplementary Note 13). In case the magnetization vector is along *z*, spin-orbit coupling further flattens this band by opening a sizeable gap *E*_SO_ ~30 meV at the crossing point, across which significant Berry curvature is concentrated (see Supplementary Note 14). It is noteworthy that while *v*_F_ has diminished dramatically, *E*_SO_ is still comparable to that of highly dispersive Dirac bands in the ferromagnetic kagome metal Fe_3_Sn_2_ (~30 meV)^13^. The regime of high spin-orbit coupling and strong electronic correlation has been pointed out as a potential parameter space to blend nontrivial band topology and interaction-driven quantum phases into a single material^63^. The kagome-stanene interaction under an inversion asymmetric environment proposes an alternative pathway to drive highly spin-orbit coupled materials towards the strong correlation regime. With the key ingredients naturally built-in, surface (or heterointerface) of FeSn, as well as isolated kagome-stanene bilayer, offers a unique physical platform to realize novel types of edge modes and correlated flat bands.

In addition to the peculiar chemical environment created by Sn and Nb:STO, the characteristic spin arrangement of FeSn (Fig. 3c) generates a spin-split band structure at the surface. Such a magnetic environment at the surface gives an opportunity to investigate how the two-dimensional band structure of the kagome-stanene bilayer changes as a function of the average sublattice magnetization. We show in Fig. 4f that the position of the DOS peak associated with the flat band in the kagome-stanene bilayer shifts to higher binding energy from *E* = −110 meV to *E* = −480 meV as average Fe spin moment (*M*_Fe_) reduces from *M*_sat_ to 0.5 *M*_sat_, where *M*_sat_ denotes Fe’s saturation moment. We attribute this as a potential origin of the temperature-dependent shift in the d*I*/d*V* peak position observed in the tunneling experiment (Fig. 3h). Gradual depolarization of *M*_Fe_ at the interface upon thermal fluctuation and the consequent reduction of the local exchange field may explain the shift from *V*_J,peak_ = −180 mV at *T* = 2 K to *V*_J,peak_ = −560 mV at *T* = 200 K. Model junction simulations taking into account thermal and dielectric effects give consistent positions of the flat band at each temperature (the observed *V*_J,peak_ is within the simulated ranges of the flat band across the entire temperature range, see Supplementary Note 7, 8, 24). By estimating *M*_Fe_ at each temperature from *V*_J,peak_, we find that the magnetic transition at the surface kagome layer effectively occurs ~316 K, reduced from the Neel temperature of FeSn extracted from bulk-sensitive measurements on single crystals (*T*_N,bulk_ ~ 365 K)^35,36,60,64^ and thin films (*T*_N,film_ ~ 358 K)^37,65^. The modulation of the flat band position as a function of the size of the spin moment suggests a possibility of engineering the chemical potential of an arbitrary magnetic kagome compound to the position of the flat band with a fine balance of thermal fluctuation, disorder, and magnetic field. It is of significant interest to study this further with, e.g., depth-resolved magnetic scattering probes or spin-resolved electron microscopes with sub-nanometer spatial resolution. Furthermore, stabilization of ultrathin FeSn, where the interface dominates the entirety of the signal, would most clearly elucidate the properties of the proposed interfacial state.

Focusing on its implication for device application, the knowledge of the surface flat band presented herein will be useful in designing heterostructure-based electronic or spintronic devices where interfacial phenomena govern the overall performance. A recent discovery of strong spin-orbit torque in *f*-electron-based flat band materials suggests that the exotic surface flat band in FeSn is also expected to generate peculiar spin-orbit torque signals^66^. When FeSn is interfaced with a conventional ferromagnet for spintronic device operations, strong Berry curvature contribution from FeSn’s surface flat band may transfer a significant spin-orbit torque to the latter, manifesting as significant anomalous Hall or Nernst signal in the second Harmonic domain^67^. In addition, if magnetic anisotropy and exchange biasing interaction of FeSn and the ferromagnet can be further engineered with appropriate chemical doping or mechanical strain, we expect that FeSn’s surface flat band will drive efficient magnetization switching in the latter^68,69^.

### Shubnikov-de Haas oscillations in FeSn/STO

**Fig. 5:**
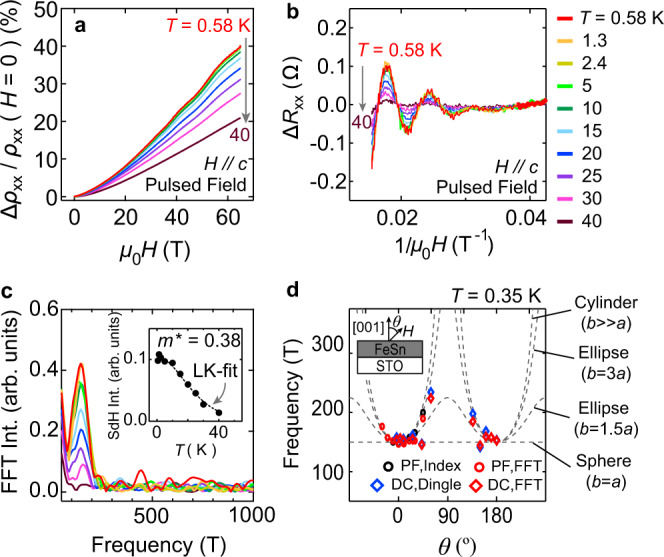
Electrical transport at high magnetic field. **a** High-field magnetoresistance of FeSn/STO at different temperatures. **b** Shubnikov-de Haas (SdH) oscillations at different temperatures, extracted from **a**. **c** Fast Fourier Transform (FFT) of the SdH oscillations in **b**. The inset shows the temperature-dependent SdH oscillation amplitudes (marker) and their fit to Lifshitz-Kosevich (LK) formula (dashed line), from which the effective mass of *m*^⋆^ = 0.38 *m*_0_ was extracted. **d** Field angle-dependent SdH oscillation frequencies. Different fitting methods were applied to data sets from experiments in two different magnet systems (see Supplementary Note 19). The overlaid dashed lines denote expected Fermi surface cross-sections assuming different Fermi pocket ellipticity. *a* and *b*, respectively, are the minor and major axes of the ellipse, also corresponding to the Fermi wavevectors along the *ab*-plane and the *c* axis of FeSn.

Finally, in order to confirm that subsurface layers of the FeSn thin films retain the band structure consistent with bulk single crystals, we performed bulk-sensitive Shubnikov-de Haas (SdH) oscillation measurements, as a complementary probe to the interface-sensitive tunneling spectroscopy. For SdH measurements, FeSn films were deposited on undoped (insulating) STO. Figure 5a shows the high-field magnetoresistance $\frac{{{\Delta }}{\rho }_{xx}}{{\rho }_{{{{{{{{{{\rm{xx}}}}}}}}}}}\left(H=0\right)}\ \equiv \ \frac{{\rho }_{{{{{{{{{{\rm{xx}}}}}}}}}}}\left(H\right)-{\rho }_{{{{{{{{{{\rm{xx}}}}}}}}}}}\left(H=0\right)}{{\rho }_{{{{{{{{{{\rm{xx}}}}}}}}}}}\left(H=0\right)}$ of an FeSn film with the thickness ${t}_{{{{{{{{{{\rm{FeSn}}}}}}}}}}}$ = 25.5 nm. The overall response is quadratic in a magnetic field with a growing magnitude from *T* = 40 K to *T* = 0.58 K, originating from enhanced electronic mobility at lower temperatures. SdH oscillations are also observed with an onset field of *μ*_0_*H* ~ 30 T. Figure 5b shows the SdH oscillations at different temperatures, from which the effective mass of *m*^⋆^ = 0.38 *m*_0_ was extracted using Lifshitz-Kosevich (LK) formula (Fig. 5c, inset). The oscillation frequency, extracted by the Fast Fourier Transformation (FFT), was *f* = 145 T (Fig. 5c). The oscillation frequency and the effective mass of the Fermi pocket are in good agreement with those of the *δ* pocket observed in FeSn bulk single crystals^22^, indicating comparable electronic structure and Fermi level position.

To investigate the geometry of the Fermi pocket, we performed field angle-dependent SdH measurements at *T* = 0.35 K. As the magnetic field is tilted away from the *c* axis, the oscillation frequency gradually increases, while its amplitude diminishes (see Supplementary Note 19). Figure 5d shows a scatter plot of the field angle-dependent oscillation frequencies extracted with various fitting methods from two independent measurements in 65 T pulsed field and 45 T DC field. The overlaid dashed lines denote expected Fermi surface cross-sections assuming different ellipticities of the Fermi pocket. The observed trend suggests a Fermi pocket highly elongated along the *c* axis with *b* ≳ 3*a*, where *a* and *b* are minor and major axes of the ellipse, respectively. The elliptical geometry of the Fermi pocket reflects the electronic hopping anisotropy in bulk FeSn, originating from its layered crystal structure (Fig. 1b). These altogether validate that subsurface layers of the FeSn film in fact retain the band structure of the bulk single-crystal FeSn with a comparable Fermi level.

## Discussion

In this work, we probed the local DOS of antiferromagnetic kagome metal FeSn at the Schottky heterointerface with an n-type degenerate semiconductor Nb:STO, using planar tunneling spectroscopy. An anomalous enhancement of the tunneling conductance ~180 meV below the Fermi level of FeSn, in conjunction with surface band structure calculations, revealed evidence for a flat band residing at the bottom-most kagome layer of FeSn at the interface. Our numerical calculations suggest that the observed surface flat band corresponds to a *d*_*z*2_ orbital derived spin-polarized flat band expected in a ferromagnetic kagome-stanene bilayer. Although our findings constitute consistent signatures of the proposed surface flat band, a critical future direction would be to directly probe the spin texture and electronic structure of the interfacial layer via space-, spin-, and layer-resolved high-resolution spectroscopy techniques. Furthermore, it is of significant interest to stabilize an isolated kagome-stanene bilayer, which would most readily facilitate the direct investigation of the surface flat band discussed herein. In connection with the degree of band flattening, the two-dimensional surface localization of a vertically elongated orbital suggests a new design principle towards flat bands with nearly zero dispersion along all directions. Viewed more broadly, these observations suggest that the surface of the magnetic flat band material, being situated in an electromagnetic environment distinct from that of the bulk, has the potential to host a flat band with unique orbital and spin characters. In addition, given the surface-localized nature of this flat band, it is expected to have a pronounced effect when embedded into heterostructure-based devices for spintronic applications.

## Methods

### Film synthesis and characterization

FeSn thin films were synthesized on single-crystal Nb-doped SrTiO_3_ (111) substrates (Shinkosha, Co. and MTI, Co.) for the tunneling measurements and on single-crystal SrTiO_3_ (111) substrates (Shinkosha, Co.) for the high-field electrical transport measurements. Prior to film synthesis, substrates were annealed at 1050 ^∘^C in air for 1 h in order to prepare atomically flat and nominally oxygen-vacancy-free surfaces. This treatment is consistent with what has been conducted in ref. ^70^. Then the substrates were loaded into MBE chamber and annealed at 600 ^∘^C in UHV for 1 h to remove any residual moisture and adsorbates from the surface. FeSn films were deposited by evaporating Fe and Sn from solid source effusion cells. For the tunneling measurements, we deposited FeSn at high-temperature *T*_g_ = 500 ^∘^C to improve the FeSn/Nb:STO interface quality. The ratio of beam-equivalent pressures (BEPs) was ${P}_{{{{{{{{{{\rm{Fe}}}}}}}}}}}{:}{P}_{{{{{{{{{{\rm{Sn}}}}}}}}}}}=1{:}2.7$, where *P*_Fe_ and ${P}_{{{{{{{{{{\rm{Sn}}}}}}}}}}}$ are BEPs for Fe and Sn, respectively. For the high-field transport measurements, the substrate temperature during deposition was *T*_g_ = 180 ^∘^C and the ratio of BEPs was ${P}_{{{{{{{{{{\rm{Fe}}}}}}}}}}}{:}{P}_{{{{{{{{{{\rm{Sn}}}}}}}}}}}=1{:}2.2$. The low-temperature-synthesized FeSn films had improved in-plane morphology compared with the high-temperature-synthesized samples. FeSn films for the transport measurements were additionally capped with amorphous BaF_2_ at *T*_g_ = 200 ^∘^C and post-annealed at *T*_g_ = 500 ^∘^C for 12 h to improve crystalline quality, all in situ in the MBE chamber. The films were characterized with an X-ray diffractometer to ensure crystalline quality and the absence of impurity phases.

### Three-terminal tunneling measurements

Tunneling measurements were carried out on five Schottky junctions consisting of Nb-doped SrTiO_3_ substrates with four different doping concentrations: 0.05 wt.%, 0.2 wt.%, 0.5 wt.%, and 0.7 wt.% (Junction #1 and #2), three of them presented in the main text. The measurements were performed with the three-terminal geometry in a Helium-4 cryostat (Quantum Design PPMS Dynacool). The tunneling contacts were made by evaporating Au/Ti electrodes onto as-grown FeSn films, using shadow masks. The current and voltage reference electrodes were made directly onto the Nb:STO substrate by removing the FeSn layer and evaporating Au/Ti. Electrical connections were made with Ag paint and gold wires. An excitation signal was generated by mixing outputs from a sinusoidal oscillator (Stanford Research model SR860) and a DC voltage source (Yokogawa model 7651). Upon applying the excitation signal to the tunneling electrode, the resulting tunnel current was measured using a lock-in amplifier (Stanford Research model SR860) and a voltmeter (Keithley model 2182a) via a current amplifier (DL Instruments model 1211). The actual voltage across the Schottky junction was monitored by measuring the potential difference between the tunnel and the reference electrodes, using a lock-in amplifier (Stanford Research model SR860) and a voltmeter (Keithley model 2182a) via voltage preamplifier (DL Instruments model 1201).

### Electrical transport measurements at high magnetic field

Electrical transport measurements were performed on two different samples at the National High Magnetic Field Laboratory. Sample #1 has a rectangular shape of ~1 mm × 2 mm and electrical contacts were made by attaching gold wires to the film top surface with Ag paint. Measurements were conducted in the 65 T pulsed magnet system in Helium-3 environment at National High Magnetic Field Laboratory (LANL). Magnetoresistance was measured in the two-terminal geometry by driving the sample with 297 *μ*A 297 kHz AC current and recording the voltage across the sample by an oscilloscope (National Instruments model 5105) through a voltage preamplifier with the gain 100. Simultaneously, the time evolution of the magnetic field pulse was monitored with a pickup coil and another oscilloscope (National Instruments model 6133). The phase-sensitive response of the sample was analyzed offline after the measurements. Sample #2 has a rectangular shape of ~2 mm × 5 mm and electrical contacts were made the same way as Sample #1. Measurements were conducted in the 45 T DC magnet system in Helium-3 environment with the standard lock-in technique at National High Magnetic Field Laboratory (Tallahassee). The non-oscillatory component of the magnetoresistance was obtained by a polynomial fit and was subtracted to get the oscillatory component. The effective mass *m*^⋆^ was obtained from the temperature dependence of the oscillation amplitude according to the LK formula $A\left(T\right)={A}_{0}\frac{2{\pi }^{2}{m}^{\star }{k}_{{{{{{{{{{\rm{B}}}}}}}}}}}T}{e\hslash {\mu }_{0}H}{\sinh }^{-1}\left(\frac{2{\pi }^{2}{m}^{\star }{k}_{{{{{{{{{{\rm{B}}}}}}}}}}}T}{e\hslash {\mu }_{0}H}\right)$, where *A*_0_ is a temperature-independent prefactor, *k*_B_ is the Boltzmann constant, and *ℏ* is the reduced Planck constant. The frequency of the oscillations was obtained by three different methods: from the slope of the extremum positions of the oscillations as a function of inverse magnetic field (index), by fast fourier transform of the oscillations as a function of inverse magnetic field (FFT), and by fitting the oscillations to the Dingle formula $A\left(T,H\right)={A}_{1}\frac{2{\pi }^{2}{m}^{\star }{k}_{{{{{{{{{{\rm{B}}}}}}}}}}}T}{e\hslash {\mu }_{0}H}\exp \left(-\frac{2{\pi }^{2}{m}^{\star }{k}_{{{{{{{{{{\rm{B}}}}}}}}}}}{T}_{{{{{{{{{{\rm{D}}}}}}}}}}}}{e\hslash {\mu }_{0}H}\right){\left(\sinh \left(\frac{2{\pi }^{2}{m}^{\star }{k}_{{{{{{{{{{\rm{B}}}}}}}}}}}T}{e\hslash {\mu }_{0}H}\right)\right)}^{-1}\sin \left(\frac{2\pi f}{{\mu }_{0}H}+\gamma \right)$, where *A*_1_ is a temperature and field-independent prefactor, *T*_D_ is the Dingle temperature, *γ* is a phase of the oscillations (Dingle).

### First-principles calculations

The FeSn slab consisted of eight crystallographic unit cells stacked along the *c*-direction and was terminated with a kagome layer on one side (*L* = 1) and a stanene layer on the other side (*L* = 8), preserving the stoichiometric bulk structure. The electronic structure of the slab was simulated with the four-component fully relativistic full-potential local-orbital (FPLO) density functional theory (DFT) code, version 18.00^71–73^. This procedure is consistent with what has been conducted in ref. ^22^. The DFT calculations were performed using the standard generalized-gradient approximation in the Perdew, Burke, and Ernzerhof (PBE)^74^ parametrization. A *k* space integration was carried out with the linear tetrahedron method using 8 × 8 × 1 subdivisions in the full Brillouin zone. Fe valence orbitals of 3*s*, 3*p*, 4*s*, 5*s*, 3*d*, 4*d*, and 4*p* and Sn valence orbitals of 4*s*, 4*p*, 4*d*, 5*s*, 6*s*, 5*d*, 5*p*, and 6*p* were used as basis states. The self-consistent electronic states were considered with ferromagnetic moments within the kagome plane with spin moments along the [100] direction (*a* axis) and antiferromagnetic ordering along the [001] direction (*c* axis). A vacuum size of 1.7924 nm was created to separate the periodic slabs. The calculations converged with a self-consistent spin density better than 10^−4^. The Fe atoms at *L* = 1 and *L* = 8 converged to 2.40 *μ*_B_ and 2.03 *μ*_B_, respectively, whereas those at inner layers converged to intermediate values between the two. Based on the converged electronic state, PYFPLO module interface of the FPLO package^71,72^ was used to derive a tight-binding Wannier Hamiltonian^75^. The basis states include atomic-orbital-like Wannier functions associated with Fe 3*d* and 4*s* states, and Sn 5*s* and 5*p* states. This Wannier model construction not only enables efficient electronic structure simulations for a thin slab but also allows the analysis of microscopic atomic on-site potentials and hopping terms.

To complement the FeSn slab simulation above, we also computed the band structure of a single Fe kagome layer, with and without a neighboring stanene layer. The DFT calculations were carried out using the Vienna ab initio simulation package^76,77^, based on the pseudopotential formalism and the Projector Augmented-Wave method^78^. The ferromagnetic and nonmagnetic calculations were converged with exchange-correlation energy functional parametrized by PBE^74^, a Γ-centered 15 × 15 × 1 Monkhorst-Pack k-mesh grid, an energy cutoff 300 eV, with or without relativistic spin-orbit coupling terms. Similarly, effective models were projected from the converged electronic state using Wannier transformations^75^ implemented in Wannier90 code^79,80^. The Wannier basis states were the Fe *d* orbitals and Sn *p* orbitals and the projected Hamiltonians give further insights into the slab electronic structure when magnetic layers were stacked.

## Supplementary information

Supplementary Information

## Data Availability

The data that support the findings of this study are available from the corresponding author on reasonable request.

## Code availability

The codes used to support the findings in this study are available from the corresponding author on reasonable request.
