# Supplementary material for: Evidence of two-dimensional flat band at the surface of antiferromagnetic kagome metal FeSn
Source: Nat Commun. 2021 Sep 15;12:5345. doi: 10.1038/s41467-021-25705-1 (PMC8443682; doi:10.1038/s41467-021-25705-1)
Supplement: Supplementary file 1 — Supplementary Information [file 41467_2021_25705_MOESM1_ESM.pdf]

# Supplementary Information:
# Evidence of two-dimensional flat band at the surface of antiferromagnetic kagome metal FeSn

Minyong Han[1,a], Hisashi Inoue[2,a,b], Shiang Fang[3], Caolan John[1], Linda Ye[1,c], Mun K. Chan[4], David Graf[5], Takehito Suzuki[1,d], Madhav Prasad Ghimire[6,7], Won Joon Cho[8], Efthimios Kaxiras[9], and Joseph G. Checkelsky[1,e]

[1]*Department of Physics, Massachusetts Institute of Technology, Cambridge, MA 02139, USA*

[2]*Frontier Research Institute for Interdisciplinary Sciences and Institute for Materials Research, Tohoku University, Miyagi 980-8577, Japan*

[3]*Department of Physics and Astronomy, Center for Materials Theory, Rutgers University, Piscataway, NJ 08854 USA*

[4]*National High Magnetic Field Laboratory, LANL, Los Alamos, NM 87545, USA*

[5]*National High Magnetic Field Laboratory, Tallahassee, FL 32310, USA*

[6]*Central Department of Physics, Tribhuvan University, Kirtipur, Kathmandu 44613, Nepal*

[7]*Leibniz Institute for Solid State and Materials Research, IFW Dresden, Helmholtzstr. 20, 01069 Dresden, Germany*

[8]*Samsung Advanced Institute of Technology (SAIT), Suwon-si, Gyeonggi-do 16678, Korea*

[9]*Department of Physics, Harvard University, Cambridge, MA 02138, USA*

[a] These authors contributed equally.

[b] Present address: National Institute of Advanced Industrial Science and Technology, Tsukuba 305-8565, Japan

[c] Present address: Department of Applied Physics, Stanford University, Stanford, California 94305, USA

[d] Present address: Department of Physics, Toho University, Chiba 274-8510, Japan

[e] checkelsky@mit.edu

## Supplementary Note 1: Three-terminal tunneling measurement

The experimental measurement circuitry for high resolution tunneling spectroscopy of FeSn / Nb:$SrTiO_3$ heterojunctions is shown in Supplementary Fig. 1. It consists of a Schottky tunneling device connected in a three-terminal geometry. A small AC excitation $U_{AC}$ is mixed with a DC bias $U_{DC}$ in a resistor-capacitor (RC) network. Here, we use a voltage divider to reduce the original oscillator output to achieve μV resolution. The three-terminal configuration minimizes the contribution from voltage drops within the semiconducting Nb:$SrTiO_3$ and helps to accurately measure the potential difference across the tunnel junction. To handle the strongly non-linear current-voltage response of the junction, we simultaneously measure the DC voltage ($V_{DC}$), DC current ($I_{DC}$), AC voltage ($dV_{AC}$), and AC current ($dI_{AC}$) responses, allowing for precise quantitative measurements of current-voltage ($I$-$V$) characteristics and differential conductance ($\frac{dI_{AC}}{dV_{AC}}$) curves of the tunnel junction.

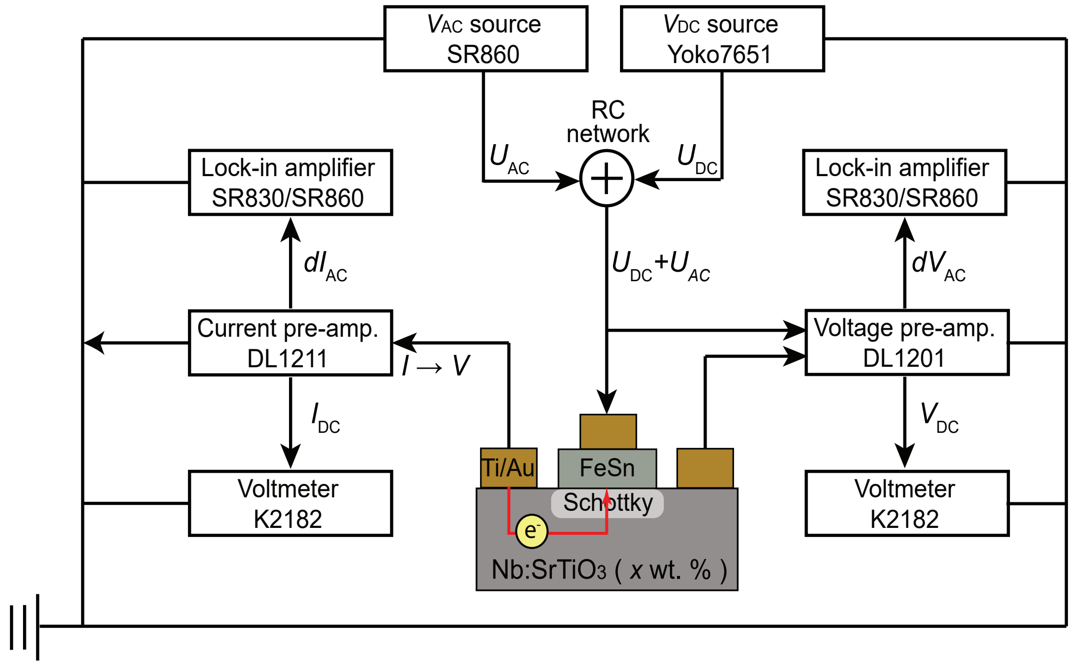


**Supplementary Figure 1 | Experimental configuration.** Schematic diagram of the three-terminal measurement configuration for FeSn / Nb:$SrTiO_3$ Schottky tunneling devices. The red arrowed line denotes the schematic conduction pathway of electrons between the tunnel and current electrodes.

**Supplementary Note 2:**

**Ti / Nb:SrTiO$_3$ ohmic contacts for the current and reference electrodes**

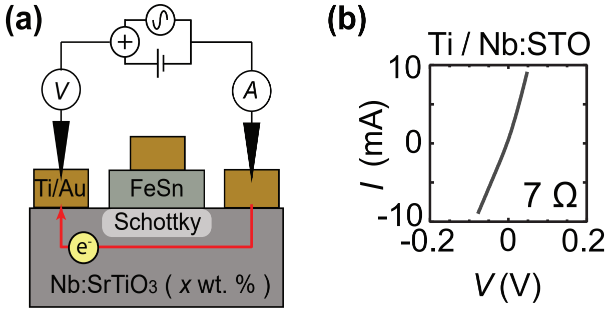


**Supplementary Figure 2 | Contact resistance characterization.** (a) Schematic drawing of the two-terminal contact resistance measurement configuration. The red arrowed line denotes the schematic conduction pathway of electrons between the reference and current electrodes. (b) Current-voltage ($I$-$V$) curve of the Ti / Nb:SrTiO$_3$ junctions.

As explained above, the three-terminal measurement scheme can extract the tunneling resistance $R_{\text{tunnel}}$ out of the two-terminal series resistance $R_{\text{series}} = R_{\text{tunnel}} + R_{\text{Nb:STO}} + R_{\text{current}}$, where $R_{\text{Nb:STO}}$ and $R_{\text{current}}$ denote the channel resistance of Nb:SrTiO$_3$ and the contact resistance of the current electrode, respectively. The three-terminal measurement is most accurate when $R_{\text{tunnel}}$ is the dominant contribution to $R_{\text{series}}$. Such a condition is met if the current electrode forms an ohmic contact with Nb:SrTiO$_3$, so that $R_{\text{tunnel}} \gg R_{\text{current}}$ and $\mathrm{d}R_{\text{current}}/\mathrm{d}V_{\text{current}}$ is constant.

Among various metals, Ti is known to form an ohmic contact with Nb:SrTiO$_3$, owing to its low work function [1]. Therefore, for the current and reference electrodes, we evaporated 10 nm-thick Ti onto bare Nb:SrTiO$_3$ and capped it with 100 nm-thick Au. Prior to the tunneling measurements, two-terminal $I$-$V$ measurements were performed between the two Ti / Nb:SrTiO$_3$ electrodes (schematic measurement configuration in Supplementary Fig. 2(a)). As shown in Supplementary Fig. 2(b), the two-terminal zero bias resistance across the two contacts was approximately 7 Ω. The experimentally measured series resistance here includes $R_{\text{series}} = R_{\text{line}} + R_{\text{Ti/Nb:STO,1}} + R_{\text{Nb:STO}} + R_{\text{Ti/Nb:STO,2}}$, where $R_{\text{line}}$ denotes the total line resistance of electronics and twisted pairs inside the cryostat. $R_{\text{line}}$ is separately calibrated to be $\sim 1-2$ Ω here and $R_{\text{Nb:STO}} \leq 1$ Ω. Therefore, the resistance of a single Ti / Nb:SrTiO$_3$ contact is estimated to be around $\sim 2-3$ Ω, several orders of magnitude more conductive than typical FeSn / Nb:SrTiO$_3$ tunnel contacts. This ensures reliable operation of the three-terminal measurement scheme.

**Supplementary Note 3: X-ray diffraction spectra of FeSn / Nb:$SrTiO_3$**

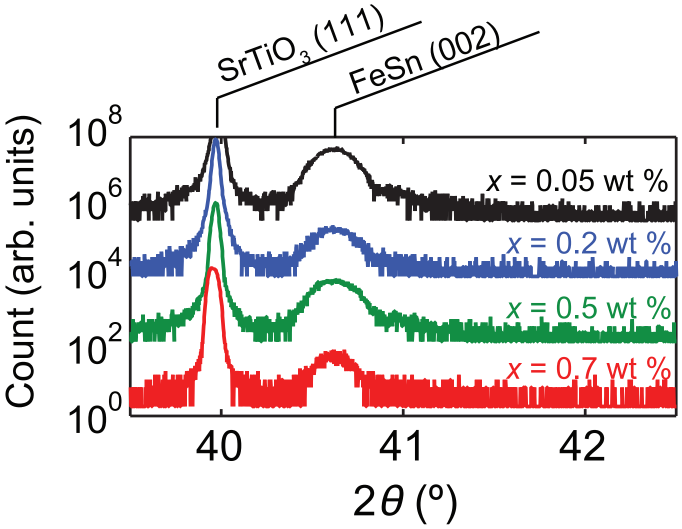


**Supplementary Figure 3 | X-ray diffraction measurements.** X-ray diffraction spectra of FeSn thin films on Nb:$SrTiO_3$ substrates with four different doping concentrations. Vertical offsets are added with respect to the $x = 0.7$ wt. % trace for clarity.

Supplementary Fig. 3 shows X-ray diffraction (XRD) spectra of FeSn / Nb:$SrTiO_3$ samples (thicknesses are 25.5 nm) with different Nb concentrations. Each curve is offset vertically with respect to the $x = 0.7$ wt. % trace for clarity. The wavelength of the incident X-ray beam was $\lambda =$ 0.154 nm. All of the XRD spectra show a dominant peak at $2\theta$ = 39.98 °, corresponding to XRD peak position of $SrTiO_3$ (111) crystallographic planes. Another broader peaks were observed in the vicinity of the substrate peak, centered around $2\theta_{\mathrm{film}}$ = 40.62 °. This peak position is close to the XRD peak position of FeSn (002) crystallographic planes $2\theta_{\mathrm{bulk}}$ = 40.52 ° expected for a bulk single crystal ($c_{\mathrm{bulk}}$ = 0.445 nm). The slight mismatch between $2\theta_{\mathrm{film}}$ and $2\theta_{\mathrm{bulk}}$ reveals small amount of in-plane tensile strain transferred from the $SrTiO_3$ (111) substrate, resulting in the compression of $c$-axis lattice constant ($\sim$ 0.2 %).

**Supplementary Note 4: *I*-*V* characteristics of FeSn / Nb:$SrTiO_3$ junctions**

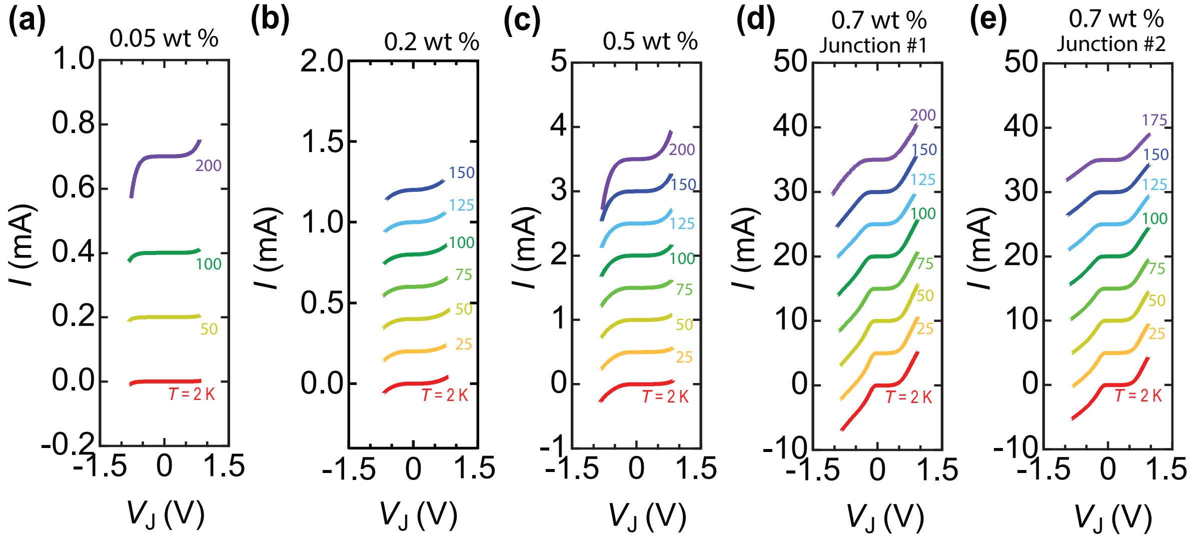


**Supplementary Figure 4 | *I*-*V* characteristics.** *I*-*V* curves measured on FeSn / Nb:$SrTiO_3$ Schottky junctions with different Nb doping concentrations measured at various temperatures. Vertical offsets are added with respect to the $T = 2$ K trace for clarity.

Supplementary Fig. 4 shows *I*-*V* characteristics at different temperatures for the FeSn / Nb:$SrTiO_3$ junctions with different Nb concentrations. Supplementary Fig. 4(b)-(d) are the junctions discussed in the main text. We show additional results in Supplementary Fig. 4(a) for $x =$ 0.05 wt. % junction and Supplementary Fig. 4(e) for $x = 0.7$ wt. % Junction #2. The $x = 0.7$ wt. % junction described in the main text is named Junction #1 here (Supplementary Fig. 4(d)). The tunnel current increases by nearly 50-fold as the doping concentration increases from $x = 0.05$ wt. % to $x = 0.7$ wt. %. Note the abrupt kink-like increase in the tunnel current under small negative $V_J$ in both $x = 0.7$ wt. % junctions. This correlates with the peak in d$I$/d$V$ around $V_J =$ -180 mV identified in the main text.

## Supplementary Note 5: d$I$/d$V$ spectra of other FeSn / Nb:$SrTiO_3$ junctions

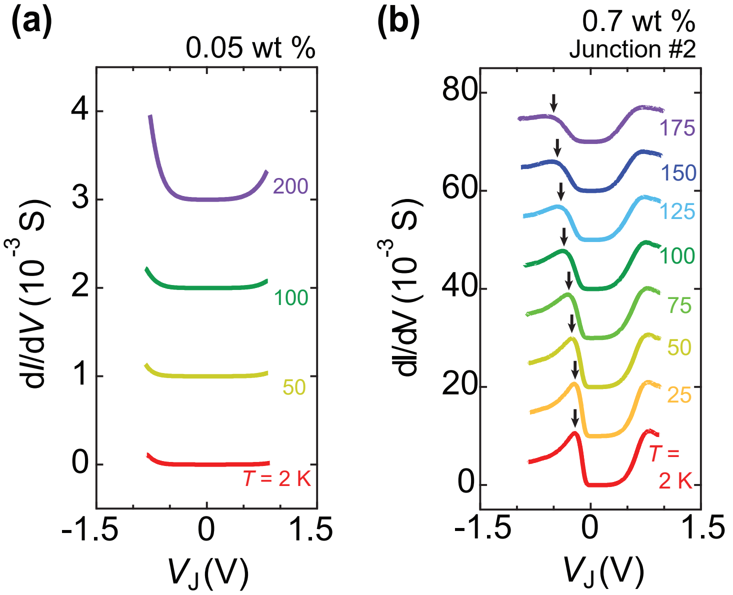


**Supplementary Figure 5 | d$I$/d$V$ characteristics.** d$I$/d$V$ curves measured on FeSn / Nb:$SrTiO_3$ Schottky junctions with different Nb doping concentrations at various temperatures. Vertical offsets are added with respect to the $T$ =2 K trace for clarity. The positions of the peak in d$I$/d$V$ are marked with arrows.

Supplementary Fig. 5 shows d$I$/d$V$ spectra at different temperatures for the FeSn / Nb:$SrTiO_3$ junctions that are not discussed in the main text. Supplementary Fig. 5(a) is from the $x = 0.05$ wt. % junction and Supplementary Fig. 5(b) is from $x = 0.7$ wt. % Junction #2. d$I$/d$V$ of the $x = 0.7$ wt. % junction #2 also shows a discernible peak at the similar energy window as in Junction #1, but with less pronounced peak sharpness. We hypothesize that the sharpness of the peak in d$I$/d$V$ may reflect the microscopic quality of the interface.

**Supplementary Note 6:**

**Signature of $SrTiO_3$ longitudinal optical phonons in the tunneling spectra**

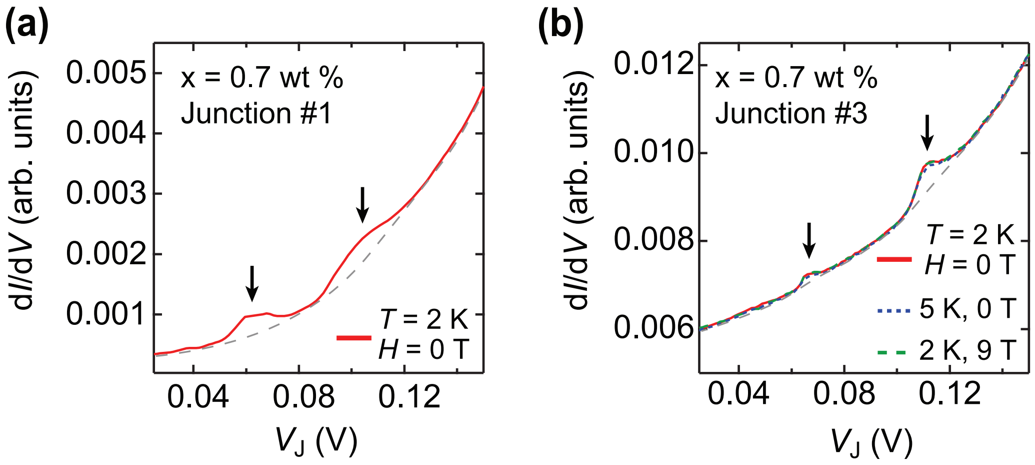


**Supplementary Figure 6 | Signature of $SrTiO_3$ longitudinal optical phonons.** d$I$/d$V$ spectra for (a) the 0.7 wt.% Junction #1 and (b) the 0.7 wt.% Junction #3. The arrows indicate the peaks arising from the electron-phonon coupling in Nb:$SrTiO_3$. The grey dashed curves are guides to the eye.

In this work, we reported a spectroscopic investigation of FeSn electronic structure with the FeSn / Nb:$SrTiO_3$ tunneling junction. Here we show that the same junction can be used to probe the electronic structure of Nb:$SrTiO_3$, owing to the fact that the tunneling conductance is dependent on the density of states (DOS) of both sides of the junction. Several previous works have reported renormalization of DOS in Nb:$SrTiO_3$ due to polaronic coupling to its highest energy and the next highest energy longitudinal optical (LO) phonons [2–5]. This manifests as replica bands below the conduction band with the energy intervals of the optical phonon modes. Supplementary Fig. 6 shows a magnification of the d$I$/d$V$ spectra of the two FeSn / Nb:$SrTiO_3$ junctions measured in this study in a positive bias range. Both junctions show peaks in the differential conductance spectra around $eV_J = 65$ meV and $eV_J = 110$ meV, which are in accordance with energies of the next highest and the highest LO phonon modes in $SrTiO_3$, respectively. Applying magnetic fields or increasing temperature does not significantly modify the peak positions. Altogether, this indicates that these peaks arise due to the electron-phonon coupling in Nb:$SrTiO_3$, and not the contributions from the FeSn layer.

**Supplementary Note 7:**

**Model calculation for the temperature dependent peak shift in the tunneling spectra**

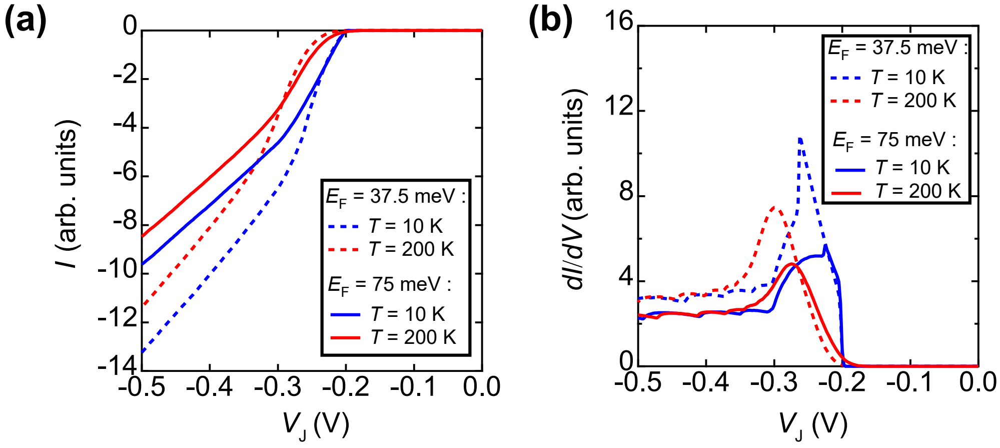


**Supplementary Figure 7 | Model Schottky junction simulations.** (a) Calculated tunnel $I$-$V$ curves and (b) d$I$/d$V$ spectra using WKB approximation for two different temperatures. The calculation assumes that a flat band exists -0.2 eV below the Fermi level. The shape of the barrier at each temperature and the electric field is determined by self-consistent Poisson equations (see Supplementary Eq. (1) below).

Experimentally we observed that the peak in the tunneling spectra shifted from $V_J$ = -0.18 V at $T$ = 2 K to $V_J$ = -0.56 V at $T$ = 200 K (see main text Fig. 3h). This suggests that the flat band position shifts to higher binding energy as temperature increases, which as a result would shift the peak in d$I$/d$V$ also at a higher temperature. In principle, the distribution of thermally activated electrons, the shape and width of the tunnel barrier, and the temperature and electric field dependence of the Schottky junction made from $SrTiO_3$ can also contribute to this.

To examine this, we calculated tunnel $I$-$V$ curves across a Schottky barrier and the corresponding d$I$/d$V$ spectra using the Wentzel–Kramers–Brillouin (WKB) approximation [6]. Here the shape of the Schottky barrier $\phi(z)$ was modeled by the self-consistent Poisson equations with the non-linear dielectric constant

$$\begin{aligned} \frac{d}{dz}\left(\varepsilon(F)\frac{d}{dz}\phi(z)\right) &= -e(n_e(z)-n_d), \\ n_e(z) &= \int D_1(E+e\phi(z))f_1(E)dE, \end{aligned} \tag{1}$$

where $n_e$ and $n_d$ are the electron and donor densities, respectively, $D_1$ and $f_1$ are the density of states and Fermi distribution functions of $SrTiO_3$, respectively, the electric-field dependent permittivity has the form $\varepsilon(F) = (1 + a/\sqrt{b + F^2})\varepsilon_0$, $\varepsilon_0$ is the electric permittivity of vacuum, and $a$ and $b$ are the temperature dependent coefficients [7]. A parabolic dispersion was assumed for $SrTiO_3$'s band structure. Tunnel current is given by

$$\begin{aligned} I(V) \sim & \iint v_{z1}(E_z) T_{1\to 2}(E_z) g(D_1(E), D_2(E+eV)) f_1(E) f_2(E+eV) dE \\ & - \iint v_{z2}(E_z) T_{2\to 1}(E_z) g(D_1(E-eV), D_2(E)) f_1(E-eV) f_2(E) dE, \end{aligned} \tag{2}$$

where $D_2$ and $f_2$ are the density of states and Fermi distribution function of FeSn, respectively, $v_{z1}$ and $v_{z2}$ are the velocities of electrons in $SrTiO_3$ and FeSn, respectively, $T_{1\to 2}$ and $T_{2\to 1}$ are the tunneling probabilities from $SrTiO_3$ to FeSn and FeSn to $SrTiO_3$, respectively, $g(D_1, D_2)$ is a function that satisfies $g(D_1, D_2) \to D_1$ for $D_2 \to \infty$ and $g(D_1, D_2) \to D_2$ for $D_1 \to \infty$, and here we used a functional form $g(D_1, D_2) = D_1 D_2/(D_1 + D_2)$. The calculated results are shown in Supplementary Fig. 7(a) for the $I$-$V$ curves and Supplementary Fig. 7(b) for the d$I$/d$V$ spectra assuming different Fermi energy of $SrTiO_3$: $E_F$ =37.5 meV (dotted line) and $E_F$ =75 meV (solid line). The flat band in FeSn was modeled as a truncated parabolic band with the bandwidth of 0.1 eV and the top band edge at -0.2 eV below the Fermi level. The DOS anomalies due to the presence of the flat band is reflected in the peak in the d$I$/d$V$ spectra, as expected. The peak positions shift to more negative energy by 0.050 eV for $E_F = 75$ meV and by 0.038 eV for $E_F$ =37.5 meV, respectively, when temperature increases from $T = 10$ K to $T = 200$ K, an order of magnitude smaller than the observed shift in the experiment. This provides evidence for the intrinsic band structure origin of the observed $\Delta V_J$, as described in the main text.

## Supplementary Note 8: Non-linear dielectric properties of $SrTiO_3$

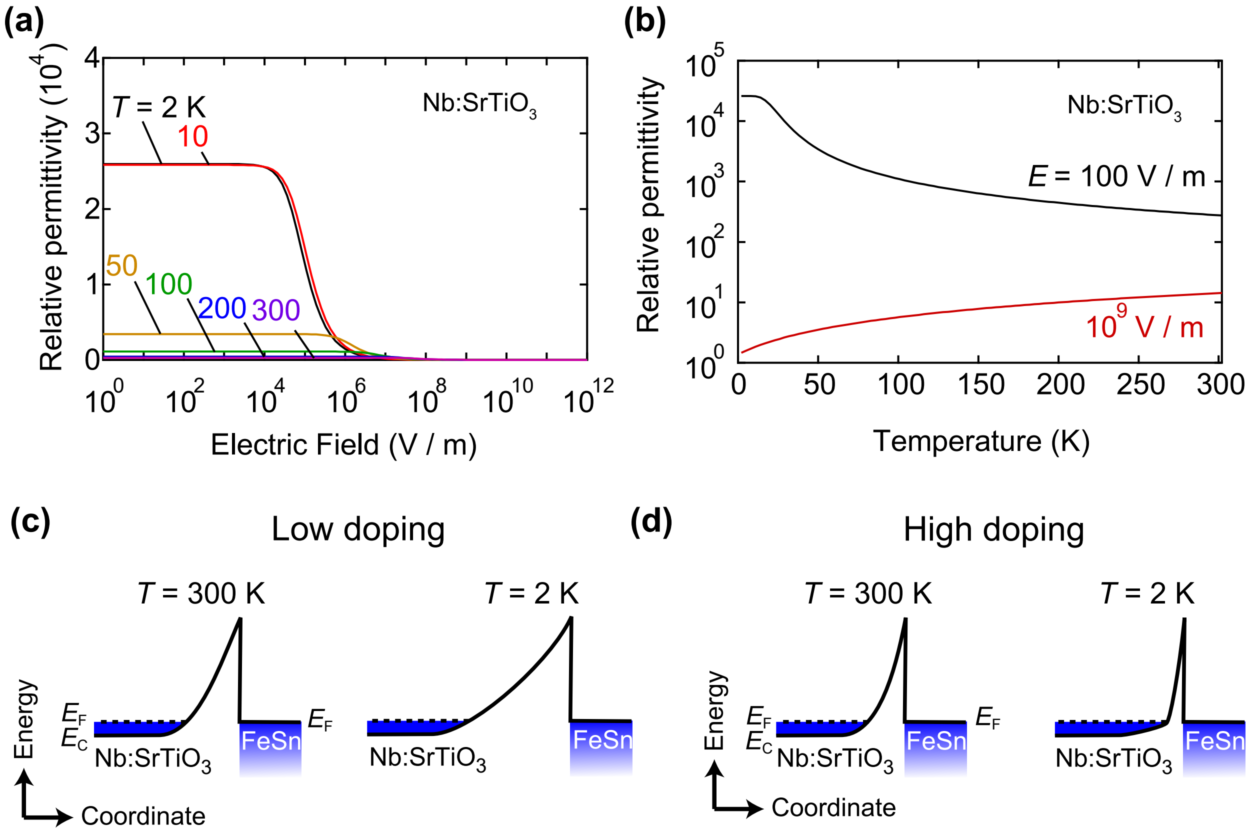


**Supplementary Figure 8 | Dielectric properties of $SrTiO_3$.** (a) Electric field dependent and (b) temperature dependent permittivity of Nb:$SrTiO_3$. (c)(d) Schematic band diagrams of the FeSn / Nb:$SrTiO_3$ Schottky junction at different temperatures for (c) low Nb doping and (d) high Nb doping.

Nb:$SrTiO_3$ has complex dielectric properties due to its proximity to a ferroelectric instability, which is suppressed by quantum fluctuations at low temperatures [8–10]. Although it remains paraelectric down to low temperature, the permittivity of Nb:$SrTiO_3$ increases by 100 times at low temperature under zero electric field up to $\varepsilon_r = 20,000$, where $\varepsilon_r$ is the relative permittivity. It is also known to exhibit a non-linear response to external and internal electric fields, which suppresses $\varepsilon_r$ drastically in a high electric field [7, 11]. These unusual dielectric properties are reflected in the electric response of the FeSn / Nb:$SrTiO_3$ Schottky tunnel junctions.

In the main text Fig. 1e, we showed that the tunnel current of the junction at low temperature exponentially grows when the Nb doping concentration in $SrTiO_3$ increases. This is due to

the cooperative action of increased screening fields in a conventional Schottky junction and the suppressed permittivity in Nb:$SrTiO_3$, both of which contribute to shortening of the depletion layer width $W_\mathrm{d} = \sqrt{\frac{2\varepsilon_\mathrm{r}\varepsilon_0\Delta\Psi_\mathrm{WF}}{eN_d}}$, where $\varepsilon_0$ is the dielectric constant of vacuum, $\Delta\Psi_\mathrm{WF}$ is the energy difference between the work function of FeSn and the electron affinity of Nb:$SrTiO_3$ ($e$ is the elementary charge and $N_\mathrm{d}$ is the Nb dopant density in Nb:$SrTiO_3$). The electric field dependence of $\varepsilon_\mathrm{r}$ of Nb:$SrTiO_3$ is shown in Supplementary Fig. 8(a) [7, 11]. Its large value under zero electric field falls off steeply to a small value across the threshold electric field $\sim 10^5$ V/m. At low doping, $W_\mathrm{d}$ takes a large value $\sim$ 100 nm owing to the large permittivity of Nb:$SrTiO_3$ at low temperature $\varepsilon_\mathrm{r} > 20,000$ (Supplementary Fig. 8(c)). However at high doping, the formation of a Schottky junction generates a large electric field in the depletion region according to $E_\mathrm{r} \sim \frac{eN_\mathrm{d}}{\varepsilon_\mathrm{r}} W_\mathrm{d}$. This electric field minimizes the permittivity down to a high field value $\varepsilon_\mathrm{r} \sim 1$ according to Supplementary Fig. 8(a), therefore further reducing the depletion layer width (Supplementary Fig. 8(d)). Note that the reduction of the depletion layer width due to the conventional Schottky mechanism alone (without non-linear suppression of permittivity) is insufficient to obtain $W_\mathrm{d}$ thin enough to perform the energy resonant tunneling spectroscopy.

The non-linear dielectricity of Nb:$SrTiO_3$ is also responsible for the non-trivial temperature dependence of the junctions. Supplementary Fig. 8(b) shows the temperature dependence of the permittivity under two different electric field strengths [7, 11]. When the electric field is small, the permittivity monotonically increases as temperature decreases. However when the electric field is large, the dependence is reversed; the high temperature permittivity is larger than the low temperature one. This suggests that the depletion layer width is thinnest at low temperature (Supplementary Fig. 8(d)), contributing to the temperature dependent sharpening of the spectral feature as in main text Fig. 3b.

## Supplementary Note 9: Semi-infinite slab calculations of antiferromagnetic FeSn

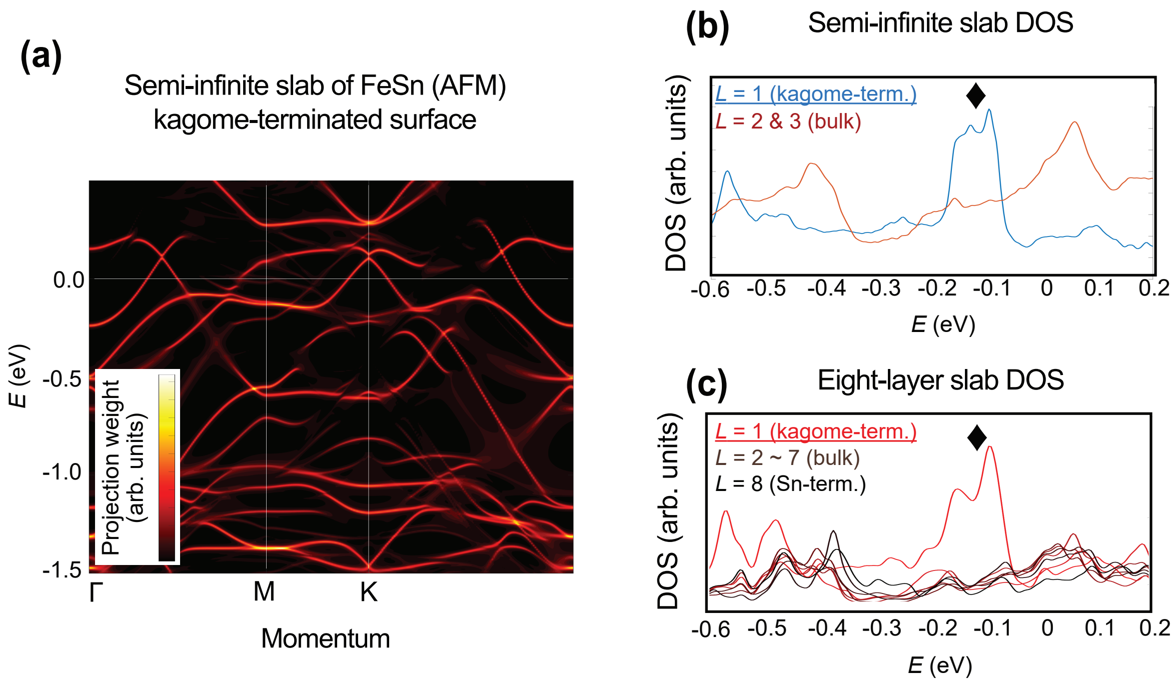


**Supplementary Figure 9 | Slab calculations with eight-layer and semi-infinite thickness.** (a) Local band structure at the kagome-terminated surface of the semi-infinite slab of FeSn. (b) The DOS spectra of the semi-infinite slab. The blue trace represents the DOS spectrum at the surface kagome layer ($L = 1$), whereas the red trace represents the average DOS spectrum of bulk kagome layers ($L = 2$ and $L = 3$). (c) The DOS spectra of the eight-layer slab. Red and black traces respectively represent the DOS spectra of the kagome-terminated surface ($L = 1$) and the Sn-terminated surface ($L = 8$). The DOS spectra of kagome layers in the bulk part are represented with graduated colors between red and black. The peaks in DOS at the kagome-terminated surface are marked with diamond markers in (b) and (c).

To verify that the band structure of the eight-layer slab of FeSn has not been affected by a finite-size effect, we investigate a semi-infinite slab of FeSn. The semi-infinite slab is designed to have the surface kagome-stanene bilayer unit cell couple with only one neighboring unit cell, while all others couple with two neighboring unit cells from above and below. Under this self-consistent construction, $L = 1$ corresponds to the kagome-terminated surface, whereas Sn-terminated surface is practically non-existent as $L \to \infty$. The computation of the surface spectral function was carried out with an iterative Green's function methods [12, 13].

Supplementary Fig. 9(a) shows the local electronic structure of the kagome-terminated surface ($L = 1$) of the semi-infinite slab. Across the entire energy and momentum, Supplementary Fig. 9(a) perfectly resembles the kagome-terminated surface band structure of the eight-layer slab presented in the main text. The identical flat band is also observed at the same energy range as in the latter. This indicates that the eight-layer slab can accurately predict the surface band structure of a realistic FeSn sample without computational artifacts.

Supplementary Fig. 9(b) and 9(c) respectively show DOS spectra of the semi-infinite layer slab and the eight-layer slab nearby the Fermi level. In Supplementary Fig. 9(b), the blue trace denotes the DOS spectrum of $L = 1$, the kagome layer at the surface, whereas the red trace denotes the average DOS spectrum of $L = 2$ and $L = 3$, the kagome layers in the bulk. In Supplementary Fig. 9(c), the layer-resolved DOS spectrum for every layer in the eight-layer slab is shown. Each layer is color-coded as follows: the kagome-terminated surface ($L = 1$) with red, the Sn-terminated surface ($L = 8$) with black, and inner layers ($2 \leq L \leq 7$) with graduated colors between red and black. We note that the peak in DOS around $E =$ -125 meV (diamonds) appears at the kagome-terminated surface ($L = 1$) in both Supplementary Fig. 9(b) and 9(c). In addition, the DOS spectra of the bulk in both Supplementary Fig. 9(b) and 9(c) also resemble each other, indicating the bulk band structure in the eight-layer slab is not deformed significantly by quantum well effect or other finite size effects.

**Supplementary Note 10: Surface-localized nature of the $d_{z2}$ orbital derived flat band**

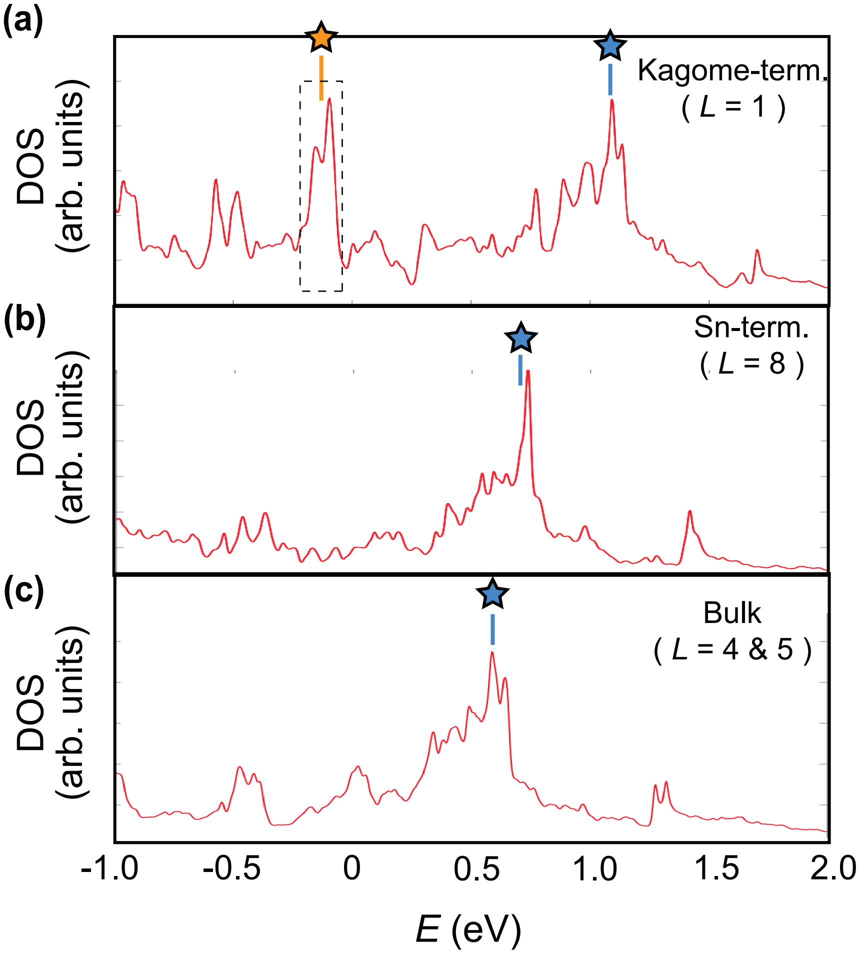


**Supplementary Figure 10 | Layer-resolved DOS spectra.** Local DOS spectra of the eight-layer slab of FeSn (a) at the kagome-terminated surface ($L = 1$), (b) at the Sn-terminated surface ($L = 8$) and (c) in the bulk. (c) is acquired by averaging the DOS of $L = 4$ and $L = 5$. Some of the pronounced features in the DOS spectra are marked with stars. Dashed line in (a) encloses the energy range of the surface flat band at the kagome-terminated surface.

Here, we present an additional evidence for the surface-localized nature of the $d_{z2}$ orbital derived flat band. Supplementary Fig. 10(a) and 10(b), respectively, show the DOS spectra of the kagome-terminated surface ($L = 1$) and the Sn-terminated surface ($L = 8$) of the eight-layer slab, while Supplementary Fig. 10(c) is the DOS spectrum at the inner part of the slab acquired by averaging the DOS at $L = 4$ and $L = 5$. Supplementary Fig. 10(a)-(c) are plotted on a wider energy range than in the main text. As guides to the eye, some of the pronounced features in the DOS spectra are marked with stars.

The DOS of the kagome-terminated surface (Supplementary Fig. 10(a)) conserves some of the noticeable features in the DOS of the bulk, though appearing at energies displaced significantly from their original positions due to distinct chemical environment at the surface. For example, the DOS peak in Supplementary Fig. 10(c) around $E \sim 600$ meV associated with the $d_{xz}/d_{yz}$ and $d_{xy}/d_{x2-y2}$ orbital derived flat band [14, 15] appears around $E \sim 1.1$ eV in Supplementary Fig. 10(a) (blue star). Unlike other features, the DOS peak around $E \sim$ -125 meV in Supplementary Fig. 10(a) is nonexistent in Supplementary Fig. 10(c) (orange star). As discussed in the main text, this peak originates from the $d_{z2}$ orbital derived flat band, which survives only at the kagome-terminated surface in the absence of translational invariance and inversion symmetry. Unlike the kagome-terminated surface, the DOS spectrum of the Sn-terminated surface (Supplementary Fig. 10(b)) more closely resembles that of the bulk, though shifted in energy by a small amount. As the kagome layer at the Sn-terminated surface is also encapsulated by two stanene layers, it is also situated in an inversion symmetric environment and therefore more closely reflects the bulk band structure.

**Supplementary Note 11:**

**Spin structure dependent band structure of kagome-stanene bilayer**

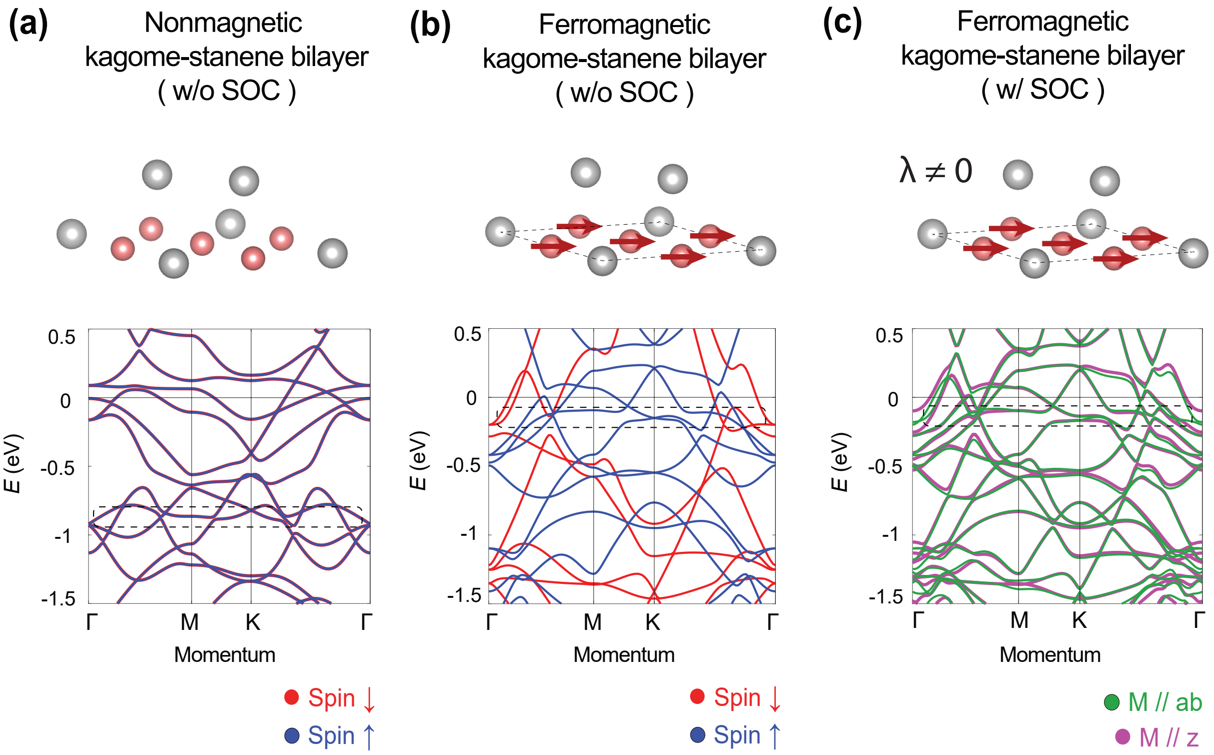


**Supplementary Figure 11 | Spin-structure dependent band structure of kagome-stanene bilayer.** Two-dimensional band structures of a kagome-stanene bilayer with (a) nonmagnetic spin texture, (b) ferromagnetic spin texture, (c) and ferromagnetic spin texture with non-zero spin-orbit coupling ($\lambda$). Red/blue bands in (a) and (b) represent spin down/up bands. Green/purple bands in (c) represent bands with spin moments within the *ab*-plane / along the *c*-axis.

Supplementary Fig. 11(a) and 11(b), respectively, show the band structures of a kagome-stanene bilayer in the nonmagnetic and ferromagnetic phases (both without spin-orbit coupling). Spin down/up bands are color-coded with red/blue, respectively. The spin degenerate $d_{z2}$ orbital derived flat band originally at $E$ = -875 meV in Supplementary Fig. 11(a) spin-splits as a result of Zeeman field and the one with the minority spin (blue) settles at $E$ = -110 meV in Supplementary Fig. 11(b) (enclosed with dashed lines). As discussed in the main text, the magnetic transition temperature at the heterointerface can be extracted by comparing this spin-splitting dependent shift in the flat band position to the temperature dependent shift in the d$I$/d$V$ peak position (see Fig. 3h and Fig. 4f in the main text). The reasonable agreement between the extracted magnetic transition

temperature and the reported Neel temperature of FeSn indicates that the magnetic ordering in the bulk persists at the heterointerface, though with reduced exchange mean field strength by a small amount.

Supplementary Fig. 11(c) shows the band structure in Supplementary Fig. 11(b) with spin-orbit coupling. The band structures with different spin moment directions are color-coded differently: spins within the *ab*-plane (green) or along the *c*-axis (purple). Spin-orbit coupling gaps out some of the band crossings, but other major features in the band structure stay nearly unchanged. In addition, the shape of each band is nearly insensitive to the spin moment direction, including that of the flat band (enclosed with dashed lines). This indicates that the spin moment direction, as determined by the magnetic anisotropy and external magnetic field, does not affect the DOS spectrum significantly.

## Supplementary Note 12:

## DOS contribution from the flat band in ferromagnetic kagome-stanene bilayers

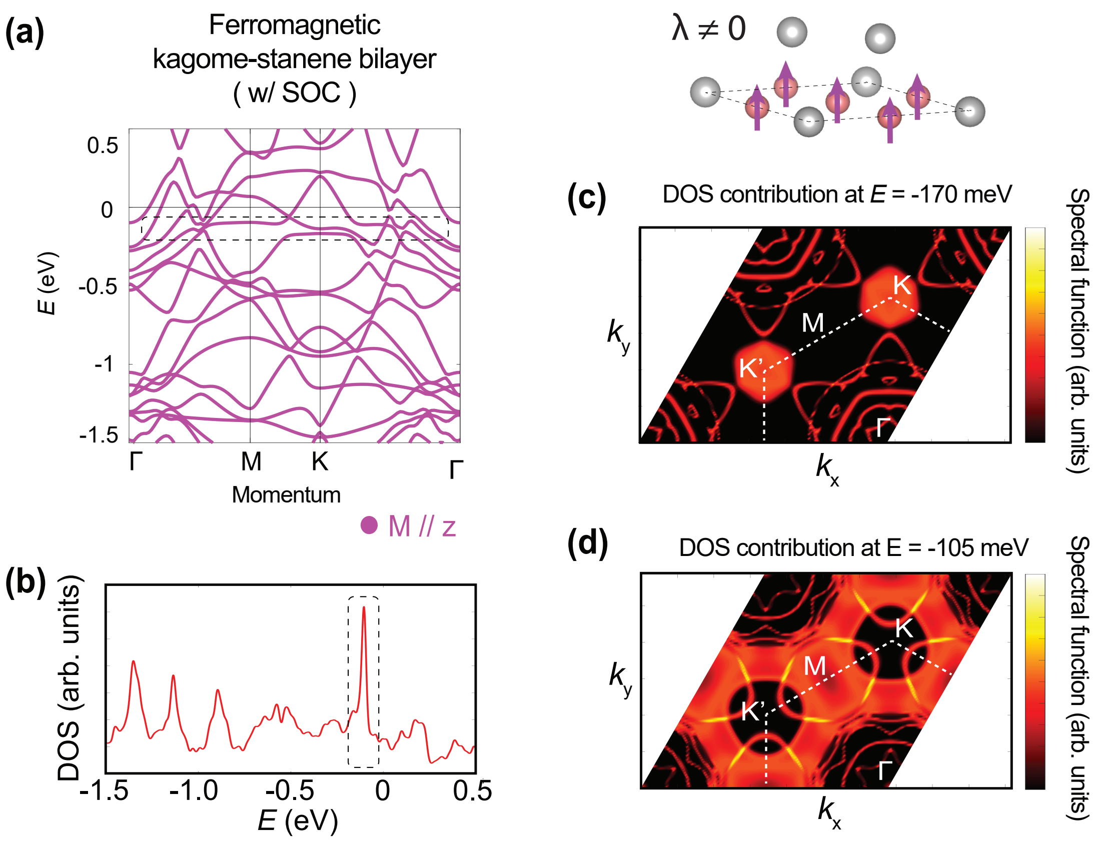


**Supplementary Figure 12 | Connection between the flat band and the DOS peak.** (a) Band structure and (b) DOS spectrum of a ferromagnetic kagome-stanene bilayer with spin moment along the *c*-axis and non-zero spin-orbit coupling. The flat band and the corresponding peak in DOS are enclosed in the dashed line in (a) and (b), respectively. Color map of DOS distribution across the Brillouin zone at (c) $E$ = -170 meV and (d) $E$ = -105 meV for the band structure in (a). Intensities in (c) and (d) correspond to spectral function amplitudes.

Here, we show more explicitly the connection between the DOS peak and the flat band. Band structure and DOS spectrum of a ferromagnetic kagome-stanene bilayer are presented in Supplementary Fig. 12(a) and 12(b). Supplementary Fig. 12(a) replots a part of Supplementary Fig. 11(c). Supplementary Fig. 12(b) is the DOS spectrum shown in the main text presented across a different energy range. Supplementary Fig. 12(c) and 12(d) show the DOS distribution across the Brillouin zone at $E$ = -170meV and $E$ = -105 meV, respectively, both of which are located within the DOS peak in Supplementary Fig. 12(b) (enclosed with dashed line). The color denotes how

much fraction of the net DOS at a given energy is contributed by the electronic states at a particular point in the Brillouin zone. Notably, a significant fraction of the DOS at $E$ = -170 meV are concentrated near the $K$-point, indicating a section of the flat band near the $K$-point is responsible for the DOS peak. Similarly, a significant fraction of the DOS at $E$ = -105 meV resides near the $M$-point, indicating a section of the flat band near the $M$-point as the origin. These confirm the flat band origin of the DOS peak at the kagome-stanene bilayer band structure.

## Supplementary Note 13:

## Electronic structure evolution with kagome-stanene interaction strength

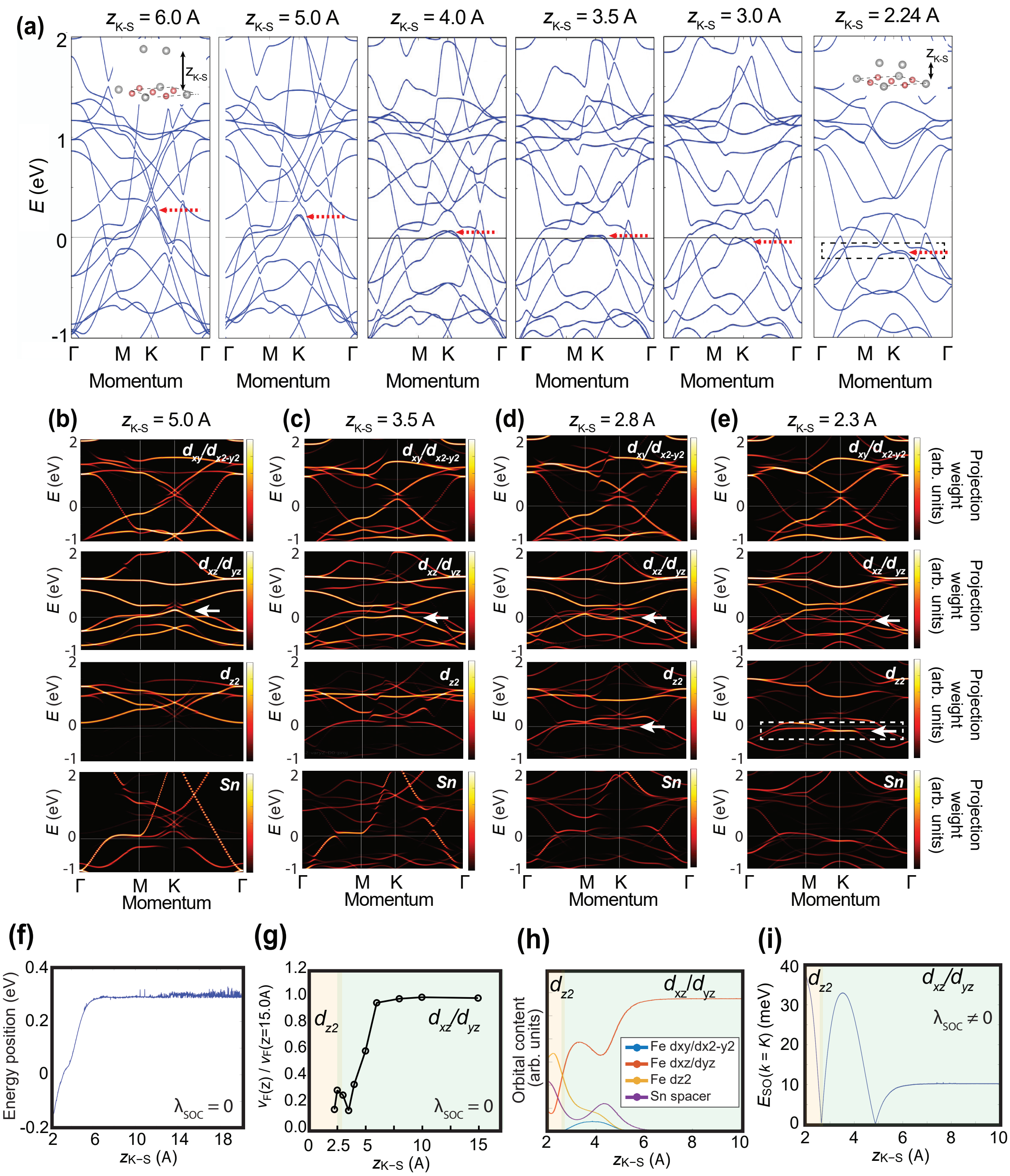


(Caption on the following page.)

**Supplementary Figure 13 | Continuous tuning of kagome-stanene interaction strength.** (a) Electronic structures of the ferromagnetic kagome-stanene bilayer with different inter-layer spacing $z_{\mathrm{K-S}}$ (only the minority spin-polarized bands are shown). Increasing $z_{\mathrm{K-S}}$ reduces the kagome-stanene interaction and the bilayer band structure continuously evolves into the isolated monolayer band structure. The red arrows mark the positions of the band crossing that is relevant to the flat band observed in the experiment. (b)-(e) Orbital decomposed kagome-stanene bilayer band structures with different $z_{\mathrm{K-S}}$. $z_{\mathrm{K-S}}$-dependent (f) energy position, (g) normalized Fermi velocity $v_{\mathrm{F}}$ (along $M$-$K$), (h) orbital composition, and (i) spin-orbit coupling induced gap size $E_{\mathrm{SO}}$ of the band crossing within the bilayer flat band.

Supplementary Fig. 13(a) shows the evolution of the ferromagnetic kagome-stanene bilayer band structure with variable kagome-stanene interaction strength. The interaction strength is controlled parametrically by the inter-layer spacing $z_{\mathrm{K-S}}$ that tunes the orbital overlap between the two layers. Supplementary Fig. 13(b)-(e) show the orbital composition of the band structures at different $z_{\mathrm{K-S}}$. Spin-orbit coupling ($\lambda_{\mathrm{SOC}}$) is not included here and only the minority spin-polarized bands are shown for clarity. When $z_{\mathrm{K-S}}$ is large (Supplementary Fig. 13(a), $z_{\mathrm{K-S}} = 6.0$ Å), the inter-layer interaction is weak and the band structure asymptotically converges to that of the nearly isolated $Fe_3Sn$ kagome monolayer. In contrast, as $z_{\mathrm{K-S}}$ approaches the actual inter-layer spacing in FeSn (Supplementary Fig. 13(a), $z_{\mathrm{K-S}} = 2.24$ Å), it represents the local band structure at the FeSn/Nb:STO Schottky heterointerface.

When $z_{\mathrm{K-S}} = 6.0$ Å, the band structure reveals several Dirac crossings at the $K$-point originating from different $d$-orbitals in the $Fe_3Sn$ kagome network, including $d_{xy}/d_{x2-y2}$, $d_{xz}/d_{yz}$, and $d_{z2}$ orbitals (Supplementary Fig. 13(b)). Here we focus specifically on one of the Dirac crossings located around $E \sim 300$ meV at $z_{\mathrm{K-S}} = 6.0$ Å (red arrow in Supplementary Fig. 13(a)). As $z_{\mathrm{K-S}}$ reduces from 6.0 Å to 2.24 Å, the position of the crossing gradually shifts down to $E = -110$ meV, the energy around which the DOS peak was observed in the tunneling experiments (Supplementary Fig. 13(a),(f)). Simultaneously, the bandwidth shrinks and the Fermi velocity near the $K$-point decreases by more than five-fold (Supplementary Fig. 13(g)). While the crossing stays robust at all $z_{\mathrm{K-S}}$ (without $\lambda_{\mathrm{SOC}}$), a significant amount of its orbital spectral weight originally concentrated in the $d_{xz}/d_{yz}$ orbital sector in the monolayer limit transfers to the $d_{z2}$ orbital sector in the bilayer limit (white arrows in Supplementary Fig. 13(b)-(e)). This demonstrates that complex interaction between the kagome layer and stanene layer and the consequent orbital hybridization are critical factors in generating the bilayer flat band.

In the presence of $\lambda_{\mathrm{SOC}}$ and the magnetization vector along $z$, a gap ($E_{\mathrm{SO}}$) opens at the crossing point, reminiscent of the gap opening in Dirac points in graphene and other systems [16, 17]. $E_{\mathrm{SO}}$ in the bilayer limit ($z_{\mathrm{K-S}} = 2.24$ Å) is larger than that in the monolayer limit ($z_{\mathrm{K-S}} \gg 2.24$ Å), as the relatively large atomic number of Sn yields a stronger $\lambda_{\mathrm{SOC}}$ in the former case. While $E_{\mathrm{SO}}$ is in general larger at smaller $z_{\mathrm{K-S}}$, the $z_{\mathrm{K-S}}$-dependence of $E_{\mathrm{SO}}$ shows a non-monotonic trend. We note that $E_{\mathrm{SO}}$ closes and reopens whenever the orbital spectral weight at the crossing point redistributes between $d_{\mathrm{xz}}/d_{\mathrm{yz}}$, $d_{\mathrm{xy}}/d_{\mathrm{x2-y2}}$, and $d_{\mathrm{z2}}$ and Sn sectors (Supplementary Fig. 13(h),(i)). In particular, such behavior is most pronounced around $z_{\mathrm{K-S}} = 2.7$ Å, the critical inter-layer spacing across which the dominant orbital character of the band crossing crossovers from $d_{xz}/d_{yz}$ to $d_{z2}$ and Sn. We hypothesize that the sign (or complex phase) of $\lambda_{\mathrm{SOC}}$ is different between these orbitals, whose competition determines the size of positive, real-valued $E_{\mathrm{SO}}$ in the resultant band structure.

**Supplementary Note 14:**

**Spin-orbit coupling induced gap and Berry curvature within the bilayer flat band**

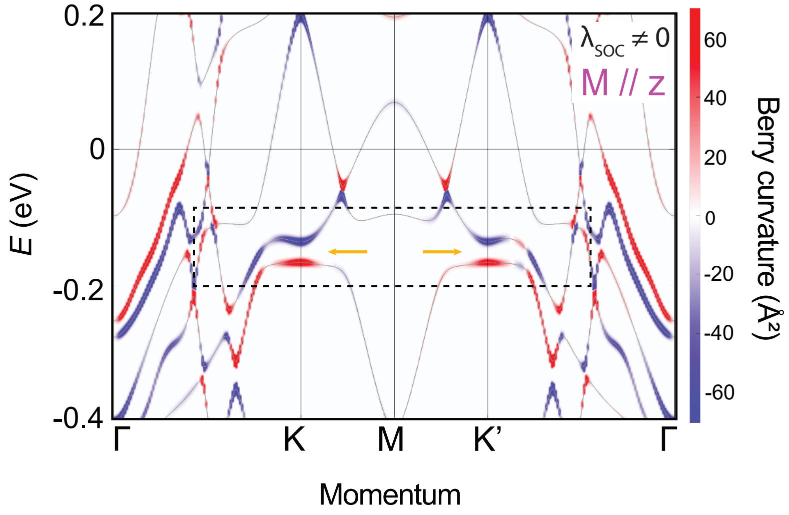


**Supplementary Figure 14 | Berry curvature analysis of the kagome-stanene bilayer flat band.** Energy- and momentum-resolved Berry curvature (BC) in the ferromagnetic kagome-stanene band structure. The magnetization vector ($M$) here is along $z$. With non-zero spin-orbit coupling, the band crossing within the kagome-stanene bilayer flat band gaps out and a sizeable BC emerges across the gap.

In the presence of spin-orbit coupling and $M$ // $z$, the band crossing at the $K$-point squeezed within the ferromagnetic kagome-stanene bilayer flat band gaps out. Supplementary Fig. 14 is the calculated Berry curvature (BC) color map of the ferromagnetic kagome-stanene bilayer band structure. The color and the brightness, respectively, denote the sign and the intensity of the BC. Significant BC is concentrated across the gap, reminiscent of the gap opening at Dirac point despite its highly suppressed dispersion.

It was discovered from a heterostructure of a conventional ferromagnet and a highly spin-orbit coupled paramagnet that an intrinsic BC contribution to the spin-orbit torque can be of comparable strength to the extrinsic spin Hall contribution [18]. Similarly, when FeSn is interfaced with a conventional ferromagnet, the BC contribution from the surface flat band is expected to transfer a strong spin-orbit torque across the interface. Minimizing the contribution from other side bands in the system will be crucial in detecting the spin-orbit torque signal purely from the flat band gap.

## Supplementary Note 15: Band structure of stanene-kagome-stanene trilayer

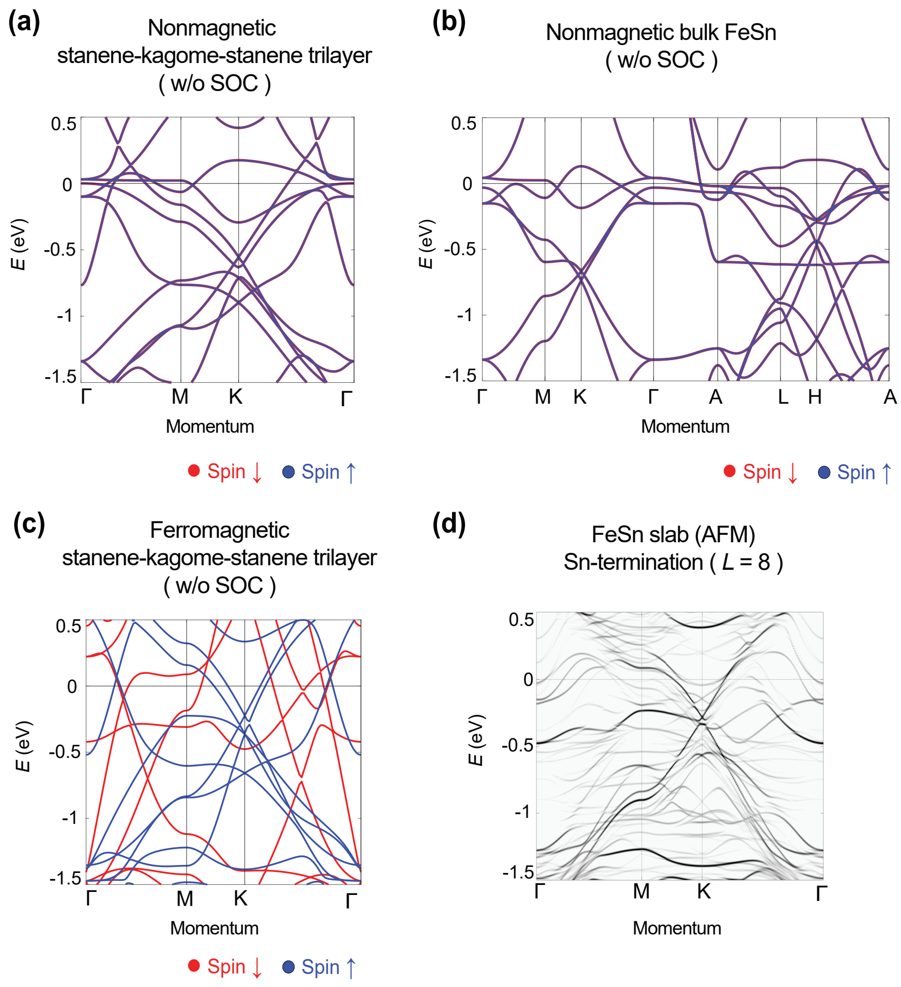


**Supplementary Figure 15 | Comparison of the trilayer band structure with the bulk band structure.** Two-dimensional band structures of stanene-kagome-stanene trilayer with (a) nonmagnetic spin texture and (c) ferromagnetic spin texture. (b) Three-dimensional band structure of bulk FeSn with nonmagnetic spin texture. (d) Local band structure of the Sn-terminated surface of the eight-layer slab. Red/blue bands in (a)-(c) represent spin down/up bands, respectively. Spin-orbit coupling is not included.

In the main text, we pointed out that the kagome layer at the kagome-terminated surface is situated in an inversion asymmetric environment, thus closely mimicking the chemical environment of the kagome-stanene bilayer. In contrast, the kagome layers in the bulk are situated in an inversion symmetric environment, which is better simulated by a stanene-kagome-stanene trilayer. In fact, we find a strong similarity between the band structure of a nonmagnetic stanene-kagome-

stanene trilayer (Supplementary Fig. 15(a)) and that of nonmagnetic bulk FeSn (Supplementary Fig. 15(b)). While some of the $d_{z2}$ orbital derived features in Supplementary Fig. 15(a) have shifted from $\Gamma - M - K - \Gamma$ plane ($k_z = 0$) to $A - L - H - A$ plane ($k_z = \pi$) in Supplementary Fig. 15(b), other bands consisting of in-plane oriented $d$ orbitals remain largely unchanged. Similarly, the kagome layer at the Sn-terminated surface is also situated in the identical chemical environment as the stanene-kagome-stanene trilayer. Supplementary Fig. 15(c) and 15(d), respectively, are the band structure of the ferromagnetic stanene-kagome-stanene trilayer and the local band structure of the Sn-terminated surface of the slab, which are in close agreement. Along with the similarity between the kagome-terminated surface and the kagome-stanene bilayer, the resemblance of the bulk and the Sn-terminated surface to the stanene-kagome-stanene trilayer also shows the importance of the chemical environment the kagome layer situates in shaping the electronic dispersion of kagome layers embedded in realistic materials.

## Supplementary Note 16: Spin texture dependent band structures of bulk FeSn

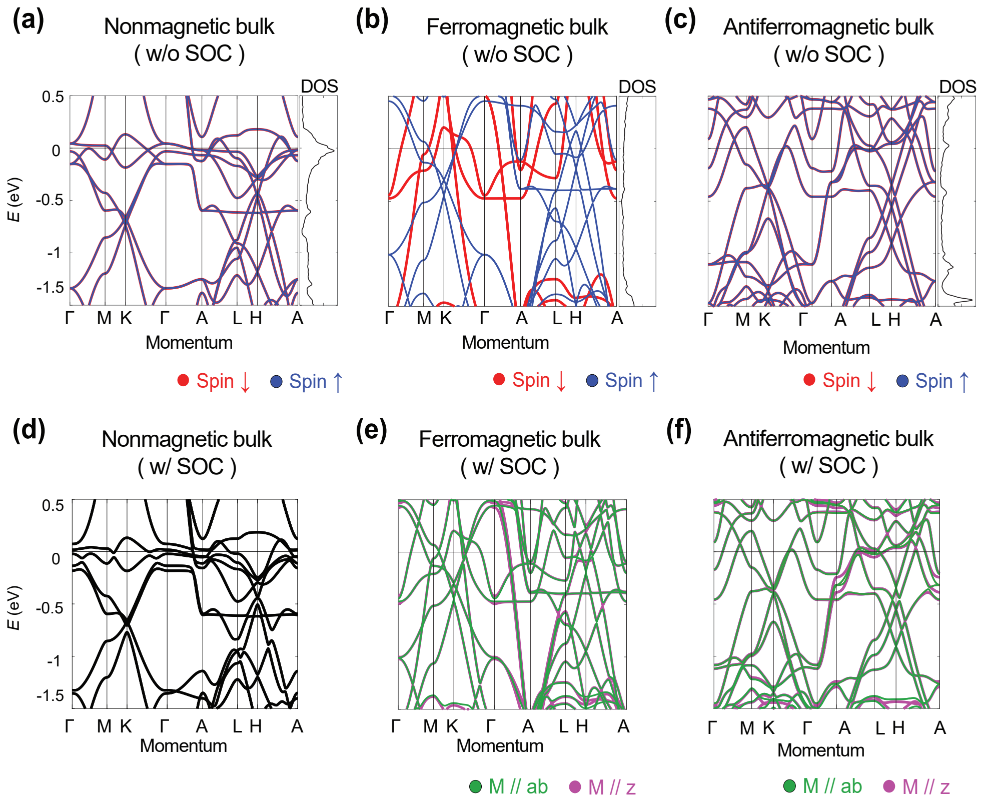


**Supplementary Figure 16 | Three-dimensional band structures of bulk FeSn.** Band structure with (a) nonmagnetic, (b) ferromagnetic, and (c) antiferromagnetic spin textures, calculated without spin-orbit coupling. Red/blue bands in (a)-(c) represent spin down/up bands, respectively. DOS spectrum for each case is presented on the side. (d)-(f) are band structures in (a)-(c), calculated with non-zero spin-orbit coupling. Green/purple bands in (e),(f) represent bands with spin moments within *ab*-plane / along the *c*-axis, respectively.

Supplementary Fig. 16(a),(b),(c) are three-dimensional band structures of nonmagnetic, ferromagnetic, and antiferromagnetic bulk FeSn, respectively, calculated without spin-orbit coupling. Spin down/up bands are color-coded with red/blue, respectively. Supplementary Fig. 16(d),(e),(f) are the band structures in Supplementary Fig. 16(a),(b),(c) calculated with spin-orbit coupling, respectively. The band structures with different spin moment directions are color-coded differently: spins within the *ab*-plane (green) or along the *c*-axis (purple). Similar to the case of kagome-stanene bilayer (Supplementary Note 11), the bulk band structures are also nearly insensitive to the spin moment direction, except in vicinity of some band crossing points.

**Supplementary Note 17:**

**Magnetic field dependent d$I$/d$V$ spectra for the $x = 0.7$ wt.% junction**

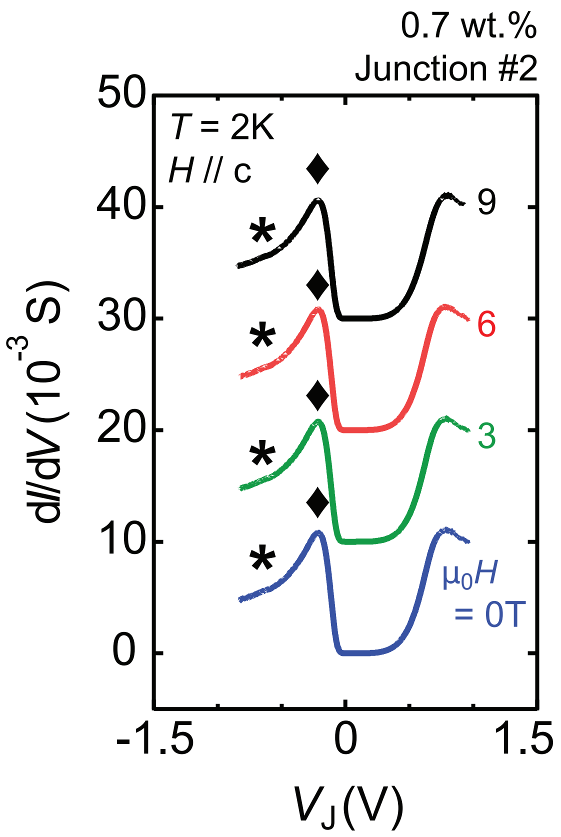


**Supplementary Figure 17 | d$I$/d$V$ characteristics under magnetic field.** d$I$/d$V$ curves for the $x = 0.7$ wt.% Junction #2 measured at different magnetic fields applied along the $c$-axis. Vertical offsets are added with respect to the $\mu_0 H = 0$ T trace for clarity. All of the measurements are conducted at $T = 2$ K. The positions of the peak and the shoulder-like features are marked with diamonds and asterisks, respectively.

Tunneling characteristics of the $x = 0.7$ wt.% Junction #2 was characterized at different magnetic fields applied along the $c$-axis: $\mu_0 H = 0, 3, 6, 9$T (Supplementary Fig. 17). We find that all of the major features in the d$I$/d$V$ spectra stay fixed in energy at all four magnetic field values, indicating the DOS has not altered with an application of magnetic field up to $\mu_0 H = 9$ T.

Magnetic susceptibility measurements on bulk single crystal FeSn have shown that Fe spins, originally pinned to $ab$-plane at $\mu_0 H = 0$ T, cant towards the $c$-axis by 0.33 $\mu_B$ with an application of $\mu_0 H \sim 50$ T along the $c$-axis [19]. The moment size of 0.33 $\mu_B$ is much smaller than Fe's saturation moment in its cousin compound $Fe_3Sn_2$ ($\sim$1.9 $\mu_B$) or that of an isolated Fe atom ($\sim$2.2

$\mu_B$) [20]. These values suggest an extremely high saturation field of FeSn ($\mu_0 H_{sat} \sim 300$ T). With $\mu_0 H \leq 9$ T, Fe spins are expected to cant out-of-plane by less than 3% of their saturation moments, thus not affecting the band structure significantly. Furthermore, as discussed previously (Supplementary Fig. 11(c), Supplementary Fig. 16(e),(f)), the band structure of FeSn is found to be insensitive to the spin moment direction, except at band crossing points. Considering the small amount of spin canting and the insensitivity of the band structure to the spin moment direction, the DOS spectra of FeSn both at the surface and in the bulk are expected to stay unchanged within $\mu_0 H \leq 9$ T, in accordance with the observed d$I$/d$V$ spectra.

## Supplementary Note 18:

## Temperature dependent Shubnikov-de Haas oscillations in other FeSn / $SrTiO_3$

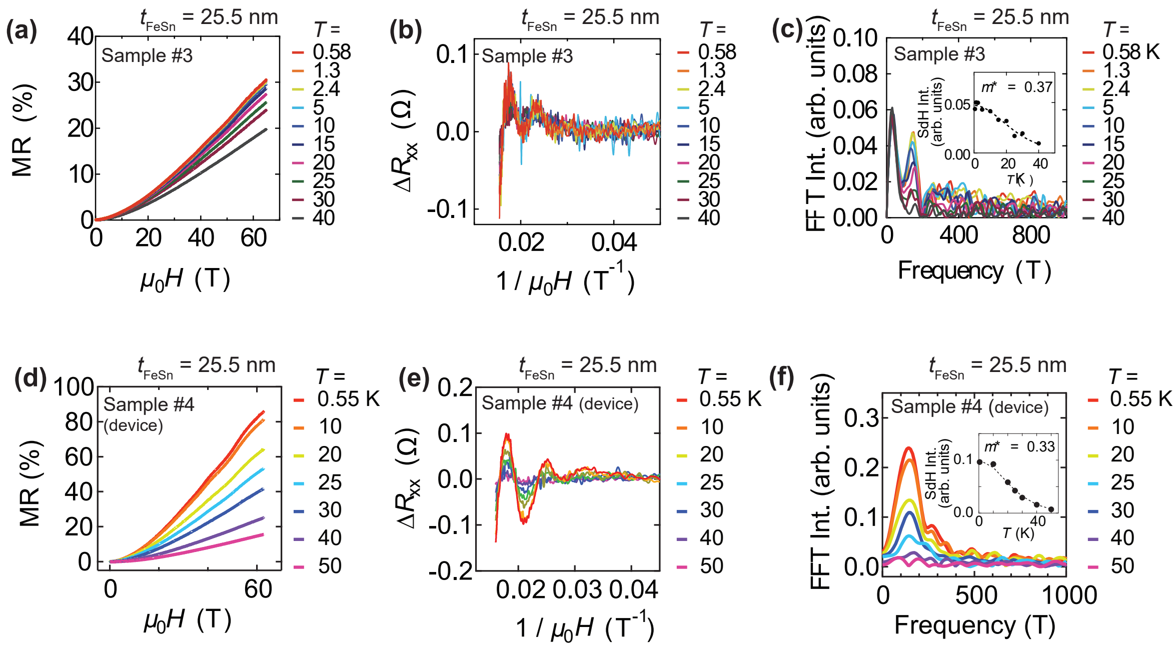


**Supplementary Figure 18 | Electrical transport measurements at high magnetic field.** (a) Magnetoresistance, (b) SdH oscillations, (c) Fourier transform, and (inset) temperature dependent oscillation amplitude measured on Sample #3. (d) Magnetoresistance, (e) SdH oscillations, (f) Fourier transform, and (inset) temperature dependent oscillation amplitude measured on Sample #4. Dashed lines in (c) and (f) are fit results to the Lifshitz-Kosevich formula.

| | $f_{FFT}$ | $m^{\star}_{LK}$ $(m_0)$ | $f_{Dingle}$ | $T_{Dingle}$ |
|---|---|---|---|---|
| Bulk $\delta$-pocket | 132 | 0.26 | N/A | 30 |
| Sample #1 | 145 | 0.38 | 153 | 34 |
| Sample #3 | 145 | 0.37 | - | - |
| Sample #4 | 143 | 0.33 | 157 | 28 |

**Supplementary Table 1 | Summary of band parameters extracted from Supplementary Fig. 18.** Bulk band parameters were extracted from Supplementary Ref. [14]. Extraction of $f_{Dingle}$ and $T_{Dingle}$ for Sample #3 was not possible due to an insufficient signal-to-noise ratio. Sample #4 was a lithographically defined device.

Supplementary Fig. 18 displays temperature dependent SdH oscillations measured from two additional FeSn / $SrTiO_3$ samples: Sample #3 (Supplementary Fig. 18(a)-(c)) and Sample #4 (Supplementary Fig. 18(d)-(f)). Supplementary Fig. 18(a),(b),(c) respectively show magnetoresistance, SdH oscillations, and Fourier transform of Sample #3 (inset in Supplementary Fig. 18(c) shows the temperature dependent SdH amplitudes and their fit to Lifshitz-Kosevich formula). Similarly, Supplementary Fig. 18(d),(e),(f) respectively show magnetoresistance, SdH oscillations, and Fourier transform of Sample #4 (inset in Supplementary Fig. 18(f) shows the temperature dependent SdH amplitudes and their fit to Lifshitz-Kosevich formula).

Sample #3 was prepared the same way as Sample #1 and Sample #2 (described in the Methods section in the main text). Sample #4 was a lithographically defined Hall bar device. The device shape was first patterned onto a film using standard photolithography, and the film area other than the Hall bar was etched away using Argon ion milling. The milling rates of $BaF_2$ capping layer and FeSn film were separately calibrated. The electrode pattern was overlaid onto the device pattern, using standard photolithography. Finally, Ti and Au were sequentially evaporated onto the contact patterns at an angle 10 $^\circ$ tilted away from the film normal direction. The purpose of this was to form a stable electrical connection between the evaporated metal and the one-dimensional edge of the film buried underneath the $BaF_2$.

The band parameters extracted from Supplementary Fig. 18(a)-(f) are summarized in Supplementary Table 1. These include oscillation frequency extracted from Fast Fourier Transform ($f_{\mathrm{FFT}}$) and Dingle fit ($f_{\mathrm{Dingle}}$), as well as effective mass ($m^\star$) and Dingle temperature ($T_{\mathrm{Dingle}}$). We include reported values of band parameters from bulk single crystal for comparison [14]. The band parameters extracted from all three films show reasonable agreement with those of the $\delta$ pocket in bulk crystals.

**Supplementary Note 19:**

**Field angle dependent Shubnikov-de Haas oscillations in FeSn / $SrTiO_3$**

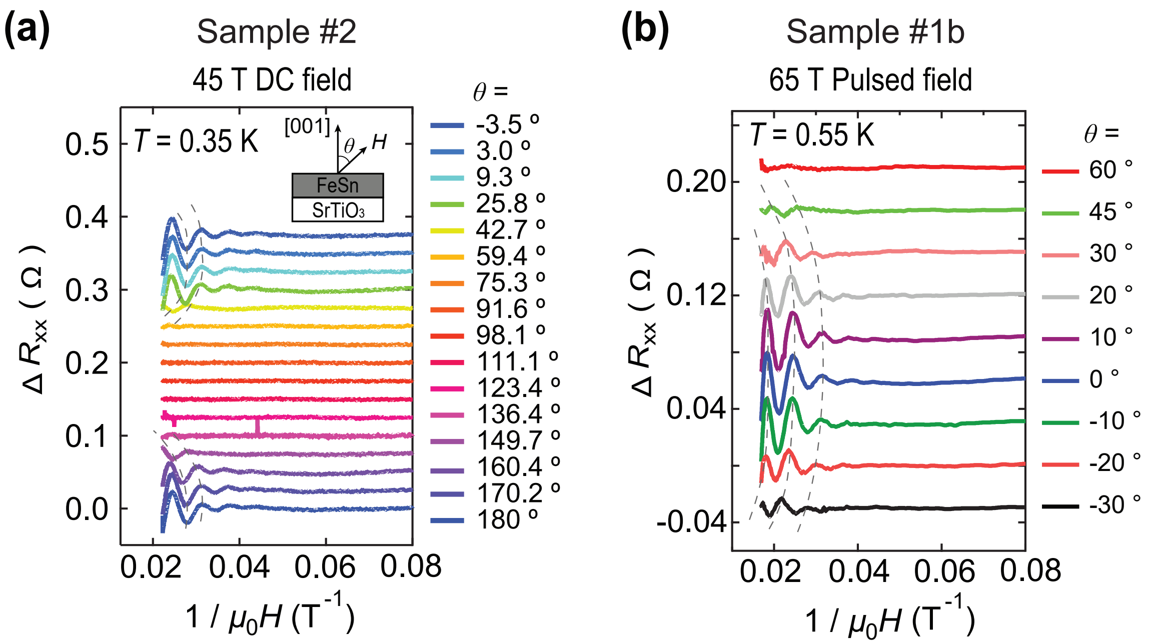


**Supplementary Figure 19 | Angle-dependent high magnetic field electrical transport measurements.** Magnetic field angle dependent SdH oscillations taken (a) from Sample #2 in the 45 T DC magnet system and (b) from Sample #1b in the 65 T pulsed magnet system. Vertical offsets are added for clarity. Dashed lines indicate the trajectories of peaks and dips in the oscillations.

Supplementary Fig. 19 shows SdH oscillations of FeSn / $SrTiO_3$ samples at different magnetic field angles $\theta$ with respect to the $c$-axis of FeSn (i.e. $H$ // $c$ corresponds to $\theta = 0\,^\circ$ or $\theta = 180\,^\circ$). Supplementary Fig. 19(a) is the data acquired from Sample #2 in the 45 T DC magnet system at $T_{\mathrm{DC}} = 0.35$ K, whereas Supplementary Fig. 19(b) is the one acquired from Sample #1b in the 65 T pulsed magnet system at $T_{\mathrm{Pulsed}} = 0.55$ K. Each curve is offset vertically by an equal amount for clarity. Note that Sample #1b is prepared by rewiring Sample #1 in the main text for four-terminal measurements. Considering the Dingle temperature of $T_{\mathrm{D}} = 34$ K for this Fermi pocket is two orders of magnitude higher than the measurement temperatures, the $\sim 0.2$ K difference between $T_{\mathrm{DC}}$ and $T_{\mathrm{Pulsed}}$ is not expected to generate any significant difference in the level broadening in the energy spectrum or $\Delta\, R_{\mathrm{xx}}/R_{\mathrm{xx}}$ in the experimental data.

As shown in Supplementary Fig. 19(a),(b), as the magnetic field tilts away from the $c$-axis, the oscillation frequency gradually increases and the oscillation amplitude is quickly suppressed. The

change in the oscillation frequency is seen clearly from the gradual shift in the peak/dip positions of the oscillations. Each peak/dip position follows an elliptical trajectory (grey dashed lines), reflecting the anisotropic Fermi pocket morphology (elongated along the *c*-axis). The oscillation frequency at each field angle was estimated by three different methods: (1) Fast Fourier transform (FFT) of the oscillations, (2) direct fitting of the oscillations to the Dingle formula, and (3) peak/dip indexing. The peak/dip indexing is performed by identifying the number of peaks/dips within a certain interval of the inverse magnetic field : $f_{\text{index}} = \left( \frac{\text{Number of oscillations}}{(1/\mu_0 H)_{\text{max}} - (1/\mu_0 H)_{\text{min}}} \right)$, where $(1/\mu_0 H)_{\text{max}}$ and $(1/\mu_0 H)_{\text{min}}$ respectively denote the maximum and minimum inverse magnetic field values at which peak/dip occurs in the oscillations.

**Supplementary Note 20: In-plane crystallographic orientation of FeSn / Nb:$SrTiO_3$**

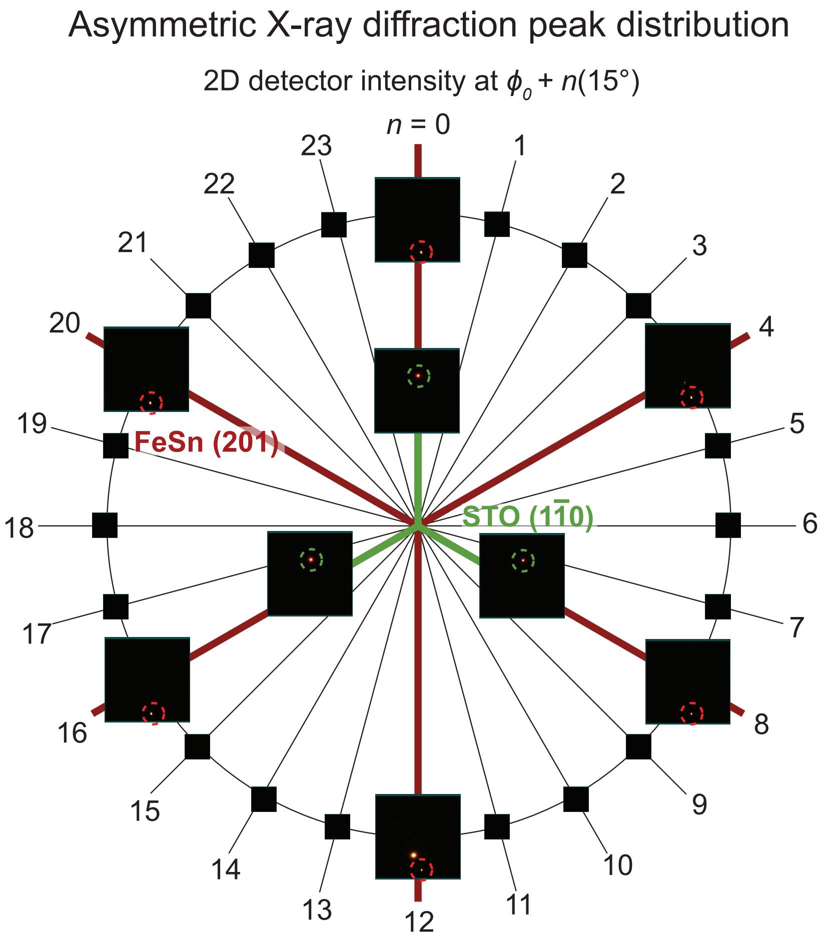


**Supplementary Figure 20 | Asymmetric X-ray diffraction measurements at different sample in-plane orientations.** Diffraction intensity maps captured from a two-dimensional detector at each angle are shown. Each image is arranged so that its position with respect to the center of the figure corresponds to the in-plane angle from which the diffraction was measured. The orientational alignment of the 6-fold symmetric FeSn (201) diffraction peaks (marked with red dashed circles) and the 3-fold symmetric $SrTiO_3$ (1$\bar{1}$0) diffraction peaks (marked with green dashed circles) reveals epitaxial locking between the two materials down to the interface.

To understand the in-plane crystallographic orientation of FeSn film with respect to that of Nb:$SrTiO_3$ substrate, we performed asymmetric X-ray diffraction measurements on a FeSn / Nb:$SrTiO_3$ sample. The wavelength of the X-ray beam was $\lambda = 0.179$ nm for this measurement. Supplementary Fig. 20 shows multiple diffraction intensity maps captured from a two-dimensional detector acquired at different sample in-plane orientations.

Across the 360° in-plane $\phi$ rotation of the sample with $2\theta$ fixed at 51.966°, we captured pronounced diffraction intensities only at discrete 6-fold symmetric $\phi$ angles (marked with red dashed circles). These angles correspond to the Bragg angles of FeSn's (201) and five other symmetry-equivalent crystallographic planes. When the $2\theta$ is changed to 37.804°, we observed a different set of 3-fold symmetric diffraction peaks exactly at the $\phi$ angles that coincide with three of the six angles where the FeSn's (201)-equivalent diffraction peaks were observed (marked with green dashed circles). They correspond to the Bragg peaks of Nb:$SrTiO_3$'s $(1\bar{1}0)$-equivalent crystallographic planes. The observation of 6-fold symmetric Bragg peaks in FeSn film confirms that the film consists of a single crystallographic domain within the plane, as well as being singly (001) oriented out-of-plane. This in-plane single-oriented nature of the film is likely enforced by the film−substrate interaction. The alignment of FeSn's (201) direction and Nb:$SrTiO_3$'s $(1\bar{1}0)$ direction reveals that the two materials are tightly bonded to each other at the interface, obeying the epitaxial relation between the two.

## Supplementary Note 21: Investigation of FeSn / Nb:$SrTiO_3$ interface morphology

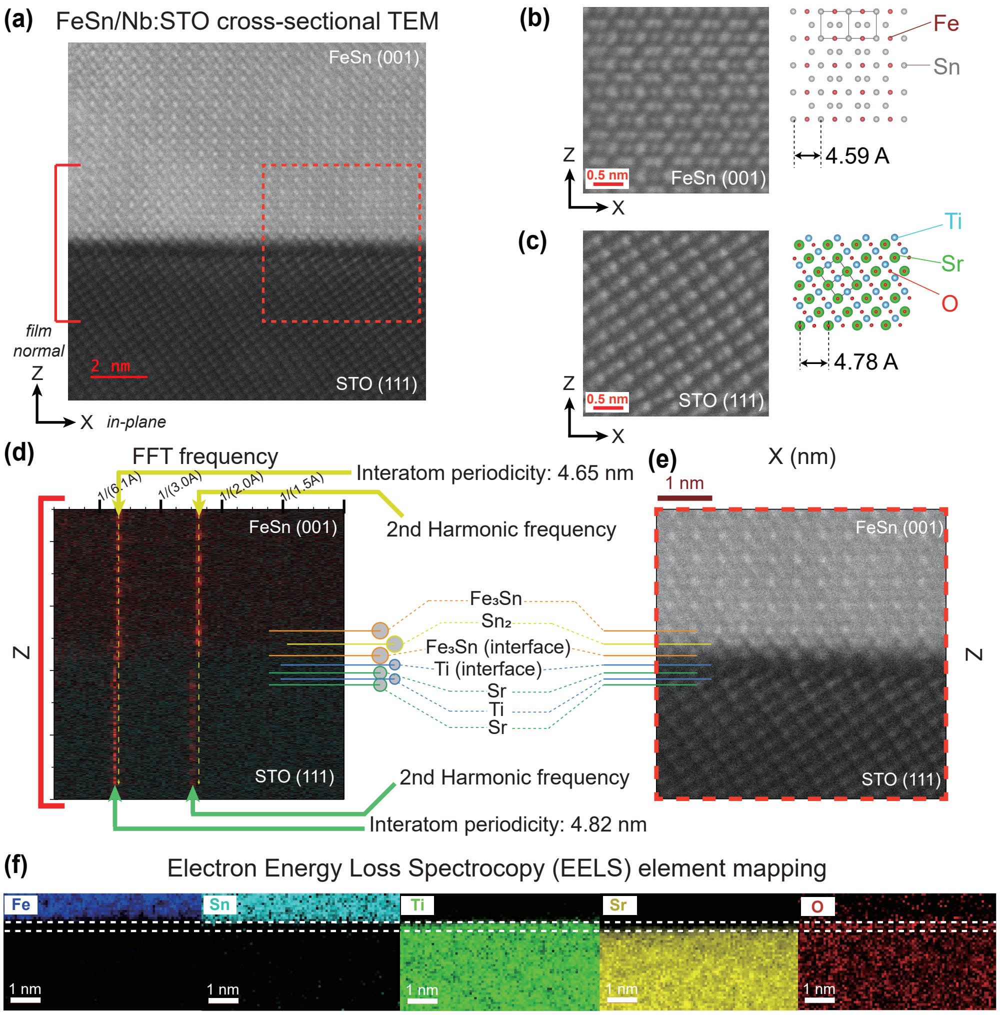


**Supplementary Figure 21 | Interface structural characterizations.** (a) Cross-sectional Transmission Electron Microscopy (TEM) image of a FeSn / Nb:$SrTiO_3$ ($x = 0.5$ wt.%) sample. The scale bar is 2 nm. Higher magnification TEM images of bulk part of (b) the FeSn film and (c) the Nb:$SrTiO_3$ substrate. The scale bars are 0.5 nm. The schematics on the right depict model atomic arrangements of (b) and (c). (d) Fast Fourier Transform (FFT) intensity colormap of the selected region in (a) (marked with the red bracket). Bright color means large intensity. (e) Magnified view of the selected region in (a) (enclosed with the red dashed line). Horizontal color-coded lines in (d) and (e) mark $Fe_3Sn$ layers (orange) and $Sn_2$ layers (yellow) in FeSn and Ti-rich layers (blue) and Sr-rich layers (green) in Nb:$SrTiO_3$ in vicinity of the interface. (f) Element-specific mapping of the region near the interface, acquired from Electron Energy Loss Spectroscopy (EELS) measurement. The scale bars are 1 nm.

To understand the detailed interface morphology of FeSn / Nb:$SrTiO_3$, we have conducted cross-sectional Transmission Electron Microscopy (TEM) measurements. The TEM measurements were performed on a different FeSn / Nb:$SrTiO_3$ sample prepared with the identical procedure as the ones we performed tunneling measurements on (see Methods in the main text). Supplementary Fig. 21(a) is a high-angle annular dark-field (HAADF) TEM image of a region near the interface. It confirms that FeSn film is highly crystalline all the way down to the interface without a polycrystalline or amorphous buffer layer. The cross-sectional views in the bulk part of FeSn film and $SrTiO_3$ substrate are shown in Supplementary Fig. 21(b) and 21(c), respectively. The observed atomic arrangements are consistent with the epitaxial relation between the two materials indicated from the asymmetric X-ray diffraction measurements (Supplementary Fig. 20).

The $Fe_3Sn$ kagome layer is found to be the preferred first-formed layer of FeSn immediately above the Ti-rich surface of Nb:$SrTiO_3$ (111). Supplementary Fig. 21(e) is a magnified view of the selected area in Supplementary Fig. 21(a) (enclosed with a red dashed line). From the magnified view, it is evident that the bottom-most $Fe_3Sn$ layer has formed above the Ti-rich layer of $SrTiO_3$ and below the $Sn_2$ layer of FeSn. Additionally, Supplementary Fig. 21(a),(e) show that the lattice strain is completely relaxed in FeSn. The inter-atom distance of the triangular network of Ti on Nb:$SrTiO_3$'s (111) surface is +3.9 % bigger than the hexagonal lattice constant of FeSn, while the observed lattice spacing in FeSn is identical to that of a strain-free bulk FeSn.

To investigate more thoroughly the exact manner through which the lattice strain is relaxed, we performed a Fast Fourier Transform (FFT) analysis for each horizontal linecut in Supplementary Fig. 21(a) and constructed a 2D colormap of FFT intensities (Supplementary Fig. 21(d)). The extracted peak frequency from the FFT analysis is expected to represent the crystal periodicity of each atomic layer. The colormap shows that the lattice strain is relaxed from the interface, showing a discontinuity in the FFT peak frequency with a 4% jump between the bottom-most $Fe_3Sn$ layer in FeSn (orange linecut) and the top-most Sr-rich layer in Nb:$SrTiO_3$ (green linecut). From the FFT peak frequencies, we acquired the corresponding interatom distances of 4.65 nm in FeSn and 4.82 nm in STO, both approximately matched with those of bulk values (Supplementary Fig. 21(b),(c)). The Ti-rich layer at the interface (blue linecut), sandwiched between the two layers with distinct lattice spacings, shows broadened intensities between two FFT frequencies, suggesting they retain crystallinity with distortions to accommodate chemical bonding between Sn and Sr typical of lattice strains relaxed at the epitaxial interface [21].

To further complement the structural analysis from TEM measurements, we also performed

Electron Energy Loss Spectroscopy (EELS) measurements. The element-specific mapping in Supplementary Fig. 21(f) shows the existence of a Ti-rich region at the interface protruding above the upper boundary of the Sr-rich region and terminating below the onset of Sn-rich region. This is in agreement with the layer-by-layer arrangement of constituent layers of FeSn and Nb:$SrTiO_3$ revealed from TEM.

The preferred formation of highly crystalline and strain-relaxed $Fe_3Sn$ kagome layer immediately above the Ti-rich surface of Nb:$SrTiO_3$ (111) is consistent with the observation of the characteristic electronic structure of the kagome-terminated surface of FeSn. (If the Sn-termination had been realized preferentially in our samples, the resultant tunneling spectra would have manifested a generally featureless d$I$/d$V$ in the $V_J < 0$ range with a small dip around $E$ = -0.28 eV, distinct from what we have measured (see Fig. 3f in the main text). The absence of any substantial buffer layer is also in accordance with highly resonant nature of the tunneling process, especially in the low temperature and high Nb-concentration regime.

**Supplementary Note 22: Fermi level pinning and its influence on Schottky band bending**

Similar to conventional metal-semiconductor junctions, different degrees of Fermi level pinning and resultant change in the barrier height can affect the tunnel conductance in FeSn / Nb:$SrTiO_3$ junctions. In the present case, the Schottky-Mott relation predicts the barrier height of FeSn / Nb:$SrTiO_3$ junction to be $\Phi_{SBH} \sim 0.5$ eV, if we use electron affinity of Nb:$SrTiO_3$ ($\chi = 4.2$ eV) and assuming the work function of FeSn is approximately similar to that of Fe ($\phi = 4.7$ eV). As for the metal-induced gap states (MIGS) in $SrTiO_3$, it is reported that the branching point is located 0.6 eV below the conduction band edge [22]. If the Fermi level is pinned to the MIGS, $\Phi_{SBH} \sim 0.6$ eV is expected, approximately similar to the value given by the Schottky-Mott relation. Increasing Nb doping concentration in STO is expected to decrease the effect of Fermi level pinning due to enhanced screening, and lead to the decrease in the barrier height and increase in the tunnel current. In the main text Figs. 1d,e, we showed an exponential growth of tunnel current across the FeSn / Nb:$SrTiO_3$ Schottky junctions as a function of Nb doping concentrations and ascribed this to the cooperative action of increased density of states in Nb:$SrTiO_3$ and shortening of the depletion layer width (see Supplementary Note 8). The suppression of Fermi level pinning effect described above can contribute additionally to this, though a significantly smaller effect is expected. While Fermi level pinning can alter the barrier shape, it does not fundamentally modify or shift the spectral features from their corresponding shapes and positions in the DOS of FeSn, regardless of the type of atomic termination at the interface. We have confirmed with TEM measurements that the interfacial layer is pristine and undeformed $Fe_3Sn$, but there remains an open possibility that the Sn-termination, if realized, would structurally reconstruct and generate a deviated DOS spectrum from what we have presented in the main text.

## Supplementary Note 23: Tunneling spectroscopy with a Schottky tunnel barrier

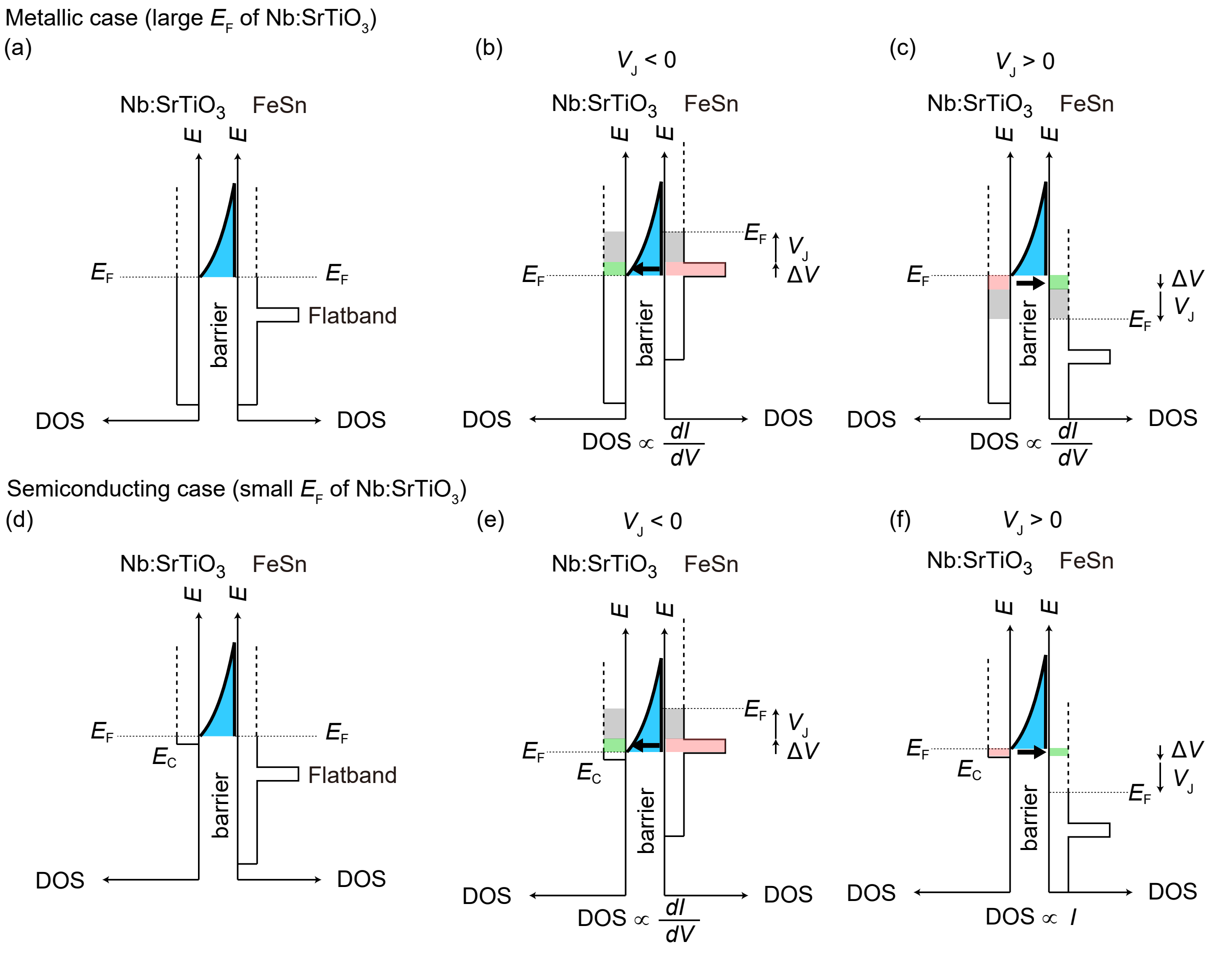


**Supplementary Figure 23 | Schematic description of electron tunneling across a Schottky barrier.** (a-c) describe spectroscopy for the case of a metallic tunneling electrode for (b) negative bias $V_J < 0$ and for (c) positive bias $V_J > 0$. (d-f) describe spectroscopy for the case of a semiconducting tunneling electrode for (e) negative bias $V_J < 0$ and for (f) positive bias $V_J > 0$.

In this study, we used semiconducting Nb:SrTiO$_3$ as the tunneling electrode for the spectroscopy and this is different from more conventional planar tunneling spectroscopy that uses a metallic electrode. We show here that under negative bias ($V_J < 0$), d$I$/d$V$ always reflects the DOS of FeSn regardless of the $E_F$ of Nb:SrTiO$_3$, because electrons tunnel from FeSn's occupied states to the unoccupied conduction band continuum of Nb:SrTiO$_3$. Interpretation of d$I$/d$V$ in the positive bias range ($V_J > 0$) can be complex in the $E_F \ll |V_J|$ regime, as described below.

The relation between DOS of FeSn and the tunneling spectra is summarized as follows:

If ($E_F$ of tunneling electrode) $\gg$ (energy range studied):

- $V_J < 0$ (tunneling from FeSn to Nb:$SrTiO_3$): DOS of FeSn corresponds to d$I$/d$V$,
- $V_J > 0$ (tunneling from Nb:$SrTiO_3$ to FeSn): DOS of FeSn corresponds to d$I$/d$V$.

If ($E_F$ of tunneling electrode) $\ll$ (energy range studied):

- $V_J < 0$ (tunneling from FeSn to Nb:$SrTiO_3$): DOS of FeSn corresponds to d$I$/d$V$ + monotonic background (present case),
- $V_J > 0$ (tunneling from Nb:$SrTiO_3$ to FeSn): DOS of FeSn corresponds to $I(V)$.

First, we consider the case of Nb:$SrTiO_3$ electrode being in the metallic regime, where $E_F$ of Nb:$SrTiO_3$ is much larger than the energy range of interest (i.e. $\gg$ 180 meV) and the DOS of Nb:$SrTiO_3$ is nearly constant in energy (Supplementary Fig. 23(a)). Under negative bias ($V_J < 0$) applied to FeSn (Supplementary Fig. 23(b)), electrons tunnel from $E < E_F$ of FeSn to the empty states in the conduction band continuum of Nb:$SrTiO_3$ above its $E_F$ (grey boxes in Supplementary Fig. 23(b)). Similarly for $V_J > 0$ (Supplementary Fig. 23(c)), electrons tunnel from $E < E_F$ of Nb:$SrTiO_3$ to the empty states at $E > E_F$ of FeSn (grey boxes in Supplementary Fig. 23(c)). Increasing or decreasing the bias voltage by $\Delta V$ induces additional tunnel current $\Delta I$ to flow from FeSn (red box Supplementary Fig. 23(b)) to Nb:$SrTiO_3$ (green box in Supplementary Fig. 23(b)) or from Nb:$SrTiO_3$ (red box Supplementary Fig. 23(c)) to FeSn (green box in Supplementary Fig. 23(c)). Since each $\Delta I$ is determined by the number of states participating additionally to the tunneling process in response to $\Delta V$, the DOS is approximately proportional to the differential conductance d$I$/d$V$.

We now consider the case of Nb:$SrTiO_3$ electrode being in the semiconducting regime, where the $E_F$ of Nb:$SrTiO_3$ is much smaller than the energy range of interest (i.e. $\ll$ 180 meV) (Supplementary Fig. 23(d)). Under negative bias ($V_J < 0$) applied to FeSn (Supplementary Fig. 23(e)), the situation is still equivalent to the case described in Supplementary Fig. 23(b) - electrons flow from $E < E_F$ of FeSn to the empty states in the conduction band continuum of Nb:$SrTiO_3$, except DOS of Nb:$SrTiO_3$ now has a finite energy dependence. We, however, note that FeSn's surface flat band generates a singular spike in its DOS, much sharper than nearly featureless DOS spectrum of Nb:$SrTiO_3$ conduction band. While the weak energy dependence of Nb:$SrTiO_3$ conduction

band may generate additional smooth background signals, we expect that the tunneling process here resembles the metallic electrode case and the d$I$/d$V$ spectra still reflects DOS of FeSn. In our tunneling experiments, we observed the enhancement of d$I$/d$V$ around $V_J = -180$ meV. Given the negative polarity of the bias voltage, we can reliably connect spectral features in d$I$/d$V$ to those in the DOS spectrum of FeSn, regardless of the $E_F$ of Nb:$SrTiO_3$ (i.e. $E_F \sim 75$ meV for $x = 0.7$ wt.%).

A caution must be taken in the positive bias range ($V_J > 0$) (Supplementary Fig. 23(f)). Electrons at $E < E_F$ of Nb:$SrTiO_3$ flow into FeSn, but the maximum number of electrons that are allowed to tunnel is bounded by the total number of electrons present in Nb:$SrTiO_3$'s conduction band (i.e. no electronic states below the conduction band edge $E_C$). In the regime $V_J > E_F$, the resonant tunneling process occurs only within a narrow energy region between the $E_C$ and $E_F$ of Nb:$SrTiO_3$, making the tunnel current $I$, rather than d$I$/d$V$, to be proportional to DOS of FeSn. $E_F$ ~75 meV (for $x = 0.7$ wt.%) situates our FeSn/Nb:$SrTiO_3$ heterostructures in the intermediate regime between the two limits described above in the $V_J > 0$ regime.

In our tunneling experiments, the d$I$/d$V$ peak at $V_J = -180$ meV was significantly broadened for the $x = 0.2$ wt.% junction (main text Fig. 3a) than for the $x = 0.7$ wt.% junction (main text Fig. 3b). This is because the Schottky barrier is thicker for smaller $x$. For a simple Schottky junction, barrier width is given by $\sqrt{2\varepsilon\Phi/eN_d}$, where $\varepsilon$ is the dielectric constant, $\Phi$ is the work function difference, $e$ is the elementary charge, and $N_d$ is the dopant density. If we use $\varepsilon = 1.50$, $\Phi = 0.7$ eV and $N_d = 6.4 \times 10^{19}$ cm$^{-3}$ (corresponding to $x = 0.2$ wt.%), the barrier width is 1.3 nm. As the barrier thickness has exponential influence on the tunneling probability, a thick barrier is not preferable and induces incoherent tunneling processes. This problem is overcome by increasing the doping concentration and consequently shortening the barrier thickness.

Viewed more broadly, tunneling spectroscopy across a Schottky heterointerface is a widely used technique for a variety of quantum materials. They include probing surface Dirac points in topological insulator thin films [23], finding absence of Mott gap at the surface of Nickelate thin films [24], measuring superconducting gap in $SrTiO_{3-x}$ [25], and identifying the band edge position of GaAs [26]. All of these reports utilized naturally formed Schottky tunnel barriers and were primarily concerned with identifying interesting band singularities at the sample surface/interface, rather than obtaining the exact shape of the DOS spectra. This technique will apply to a wide class of quantum materials containing charge neutral points, flat bands, van Hove singularities, or interaction-induced gaps.

## Supplementary Note 24: Comparing d$I$/d$V$ simulation and experiment

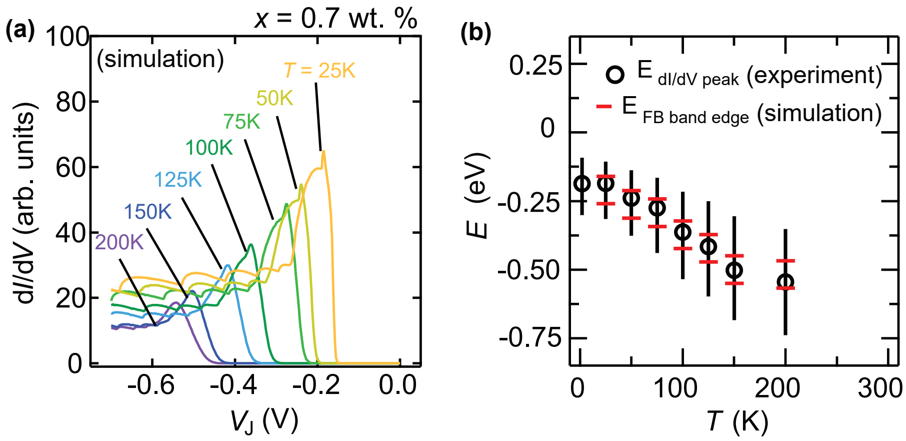


**Supplementary Figure 24 | Model Schottky junction simulations with $x = 0.7$ wt. %.** (a) Calculated d$I$/d$V$ spectrum at each temperature. The flat band positions are adjusted so as to match the experimental d$I$/d$V$ peak positions. (b) Simulated top/bottom band edges of the flat band (red horizontal bars) overlaid onto experimental d$I$/d$V$ peak positions (black circles) and their full width at half maximum (black vertical bars).

| | $E_{\mathrm{dI/dV}}$ (meV) experiment | $E_{\mathrm{FB,top}}$ (meV) simulation | $E_{\mathrm{FB,bottom}}$ (meV) simulation |
|---|---|---|---|
| 25K | -185 | -160 | -260 |
| 50K | -239 | -212 | -312 |
| 75K | -275 | -243 | -343 |
| 100K | -363 | -323 | -423 |
| 125K | -416 | -372 | -472 |
| 150K | -503 | -450 | -550 |
| 200K | -560 | -468 | -568 |

**Supplementary Table 2 | Summary of band parameters extracted from experiments and simulations.** Top (middle column) and bottom (right column) edges of the simulated flat band at each temperature in comparison with experimentally observed d$I$/d$V$ peak positions (left column).

In Supplementary Note 23, we described that d$I$/d$V$ in the $V_J < 0$ regime does reflect the DOS

information of FeSn. Using the methodology presented in Supplementary Note 7, we performed junction simulations in the $V_J < 0$ regime at each temperature and extracted the estimate of the actual flat band positions from the observed d$I$/d$V$ peak positions. The $E_F$ of Nb:$SrTiO_3$ electrode was assumed to be 75 meV (i.e. $x = 0.7$ wt.%) and the flat band was assumed to have a parabolic dispersion and a bandwidth of 0.1 eV, similar to the setup in Supplementary Note 7. Iterative processes were taken at each temperature: simulate a d$I$/d$V$ spectrum at a given temperature with the flat band at an arbitrary energy, compare with the experimental d$I$/d$V$ spectrum at that temperature, and simulate again with modified flat band position. Simulation-experiment comparisons were conducted repeatedly until their peak positions matched reasonably (Supplementary Fig. 24(a)). The simulations took into account the semiconducting nature of Nb:$SrTiO_3$, its complex dielectric effects, and other thermal energy induced shift and broadening of spectral features. While small corrections did occur, they were negligible and the experimental d$I$/d$V$ peak positions are within the simulated top and bottom edges of the flat band, indicating the experimental d$I$/d$V$ peak position is a good indicator of the flat band position (Supplementary Fig. 24(b) and Supplementary Table 2).

[1] Park, C., Seo, Y., Jung, J. & Kim, D.-W. Electrode-dependent electrical properties of metal/Nb-doped $SrTiO_3$ junctions. *J. Appl. Phys.* **103**, 054106 (2008).

[2] Swartz, A. G. *et al.* Polaronic behavior in a weak-coupling superconductor. *Proc. Natl. Acad. Sci. U.S.A.* **115**, 1475 (2018).

[3] Chen, C., Avila, J., Frantzeskakis, E., Levy, A. & Asensio, M. C. Observation of a two-dimensional liquid of Fröhlich polarons at the bare $SrTiO_3$ surface. *Nat. Commun.* **6**, 8585 (2015).

[4] Wang, Z. *et al.* Tailoring the nature and strength of electron–phonon interactions in the $SrTiO_3$ (001) 2D electron liquid. *Nat. Mater.* **15**, 835 (2016).

[5] Boschker, H., Richter, C., Fillis-Tsirakis, E., Schneider, C. W. & Mannhart, J. Electron—phonon coupling and the superconducting phase diagram of the $LaAlO_3$—$SrTiO_3$ interface. *Sci. Rep.* **5**, 12309 (2015).

[6] Simmons, J. G. Generalized formula for the electric tunnel effect between similar electrodes separated by a thin insulating film. *J. Appl. Phys.* **34**, 1793 (1963).

[7] Susaki, T., Kozuka, Y., Tateyama, Y. & Hwang, H. Y. Temperature-dependent polarity reversal in Au/Nb:$SrTiO_3$ Schottky junctions. *Phys. Rev. B* **76**, 155110 (2007).

[8] Müller, K. A. & Burkard, H. $SrTiO_3$: An intrinsic quantum paraelectric below 4 K. *Phys. Rev. B* **19**, 3593 (1979).

[9] Sakudo, T. & Unoki, H. Dielectric properties of $SrTiO_3$ at low temperatures. *Phys. Rev. Lett.* **26**, 851 (1971).

[10] Weaver, H. E. Dielectric properties of single crystals of $SrTiO_3$ at low temperatures. *J. Phys. Chem. Solids* **11**, 274 (1959).

[11] Yamamoto, T. *et al.* Effect of the field dependent permittivity and interfacial layer on $Ba_{1-x}K_xBiO_3$/Nb-doped $SrTiO_3$ Schottky junctions. *Jpn. J. Appl. Phys.* **36**, L390 (1997).

[12] Sancho, M. P. L., Sancho, J. M. L. & Rubio, J. Quick iterative scheme for the calculation of transfer matrices: application to Mo (100). *J. Phys. F: Met. Phys.* **14**, 1205 (1984).

[13] Sancho, M. P. L., Sancho, J. M. L. & Rubio, J. Highly convergent schemes for the calculation of bulk and surface Green functions. *J. Phys. F: Met. Phys.* **15**, 851 (1985).

[14] Kang, M. *et al.* Dirac fermions and flat bands in the ideal kagome metal FeSn. *Nat. Mater.* **19**, 163 (2020).

[15] Kang, M. *et al.* Topological flat bands in frustrated kagome lattice CoSn. *Nat. Commun.* **11**, 4004 (2020).

[16] Kane, C. L. & Mele, E. J. Quantum spin Hall effect in graphene. *Phys. Rev. Lett.* **95**, 226801 (2005).

[17] Haldane, F. D. M. Model for a quantum Hall effect without Landau levels: condensed-matter realization of the "parity anomaly.". *Phys. Rev. Lett.* **61**, 2015 (1988).

[18] Kurebayashi, H. *et al.* An antidamping spin–orbit torque originating from the Berry curvature. *Nat. Nanotechnol.* **9**, 211 (2015).

[19] Kakihana, M. *et al.* Electronic states of antiferromagnet FeSn and Pauli paramagnet CoSn. *J. Phys. Soc. Jpn.* **88**, 014705 (2019).

[20] Ye, L. *et al.* Massive Dirac fermions in a ferromagnetic kagome metal. *Nature* **555**, 638 (2018).

[21] Springholz, G. Surface modifications due to strain relaxation in lattice-mismatched heteroepitaxy. In *Advances in Solid State Physics 35*, 277–302 (Springer, Berlin, 1996).

[22] Kumar, V. S. & Niranjan, M. K. Precise control of Schottky barrier height in $SrTiO_3$/$SrRuO_3$ heterojunctions using ultrathin interface polar layers. *J. Phys. D Appl. Phys.* **49**, 255302 (2016).

[23] Yoshimi, R. *et al.* Dirac electron states formed at the heterointerface between a topological insulator and a conventional semiconductor. *Nat. Mater.* **13**, 253 (2014).

[24] Kozuka, Y., Susaki, T. & Hwang, H. Y. Rectifying $NdNiO_3$ / Nb:$SrTiO_3$ junctions as a probe of the surface electronic structure of $NdNiO_3$. *Appl. Phys. Lett.* **88**, 142111 (2006).

[25] Binnig, G. & Hoenig, H. Energy gap of the superconducting semiconductor $SrTiO_{3-x}$ determined by tunneling. *Solid State Commun.* **14**, 597 (1974).

[26] Conley, J. W. & D., M. G. Tunneling Spectroscopy in GaAs. *Phys. Rev.* **161**, 681 (1967).
